# Synthesis, Spectral and Solid State Characterization of a New Bioactive Hydrazine Bridged Cyclic Diphosphonium Compound

**DOI:** 10.3390/molecules17032567

**Published:** 2012-03-02

**Authors:** Milica Milenković, Beata Warżajtis, Urszula Rychlewska, Dušanka Radanović, Katarina Anđelković, Tatjana Božić, Miroslava Vujčić, Dušan Sladić

**Affiliations:** 1 Faculty of Chemistry, University of Belgrade, Studentski trg 12-16, 11000 Belgrade, Serbia; 2 Faculty of Chemistry, A. Mickiewicz University, Grunwaldzka 6, 60-780 Poznań, Poland; 3 Institute of Chemistry, Technology and Metallurgy, University of Belgrade, Njegoševa 12, P.O. Box 815, 11000 Belgrade, Serbia

**Keywords:** hydrazine bridged diphosphonium compound, DNA cleavage, brine shrimp test, X-ray

## Abstract

The facile preparation of a racemic hydrazine bridged diphosphonium compound possessing a ring system analogous to bicyclo[3.3.2]decane is reported. Although the reaction yield is low, the structure of the compound, which possesses an eight-membered ring, two phosphonium cationic centers, a biimino bridge, molecular chirality and two fused aromatic rings locked into roughly perpendicular planes is unusual. The compound displays substantial biological activity in the brine shrimp test and cleaves plasmid DNA.

## 1. Introduction

A major interest of our research group is devoted to metal complexes of polydentate dihydrazone ligands [1,2,3,4,5], mainly because some of these complexes display substantial cytotoxic activity [2,3,4]. While searching for new ligands we have focused our attention on those that combine both hard and soft donor atoms [6,7,8,9,10,11], in particular the heterofunctionalised phosphines, where the phosphorus is the soft donor, and the hard donor is either an oxygen or nitrogen atom. Complexes of Pt(II), Pd(II), and Ni(II) with the condensation derivative of 2-(diphenylphosphino)benzaldehyde and semioxamazide have been described in the literature as compounds possessing antibacterial and antifungal activity [12] and the Pd(II) complex of the condensation product of 2-(diphenylphosphino)benzaldehyde and ethyl hydrazinoacetate has been shown to exhibit anticancer activity [13].

Following these findings, we set out to prepare a condensation product of 2-(diphenylphosphino)-benzaldehyde and malonic acid dihydrazide and its corresponding metal complexes. Since the synthesis of the desired ligand was unsuccessful at pH ~ 4, more acidic conditions were applied. Moreover, a templated synthesis in the presence of the cadmium(II) and cobalt(II) ions was attempted. Surprisingly, in all cases a biimino bridged diphosphonium compound was obtained. In this paper, the synthesis, characterization and preliminary biological activity of this newly obtained compound are reported.

## 2. Results and Discussion

### 2.1. Synthesis

In order to synthesize the condensation product of 2-(diphenylphosphino)benzaldehyde and malonic acid dihydrazide, we have investigated the reaction of these compounds under diverse conditions. The optimum pH for this kind of condensation is typically about 4 [1], but our attempts to synthesize the desired compound at this pH were unsuccessful, and the starting materials were recovered.

**Scheme 1:**
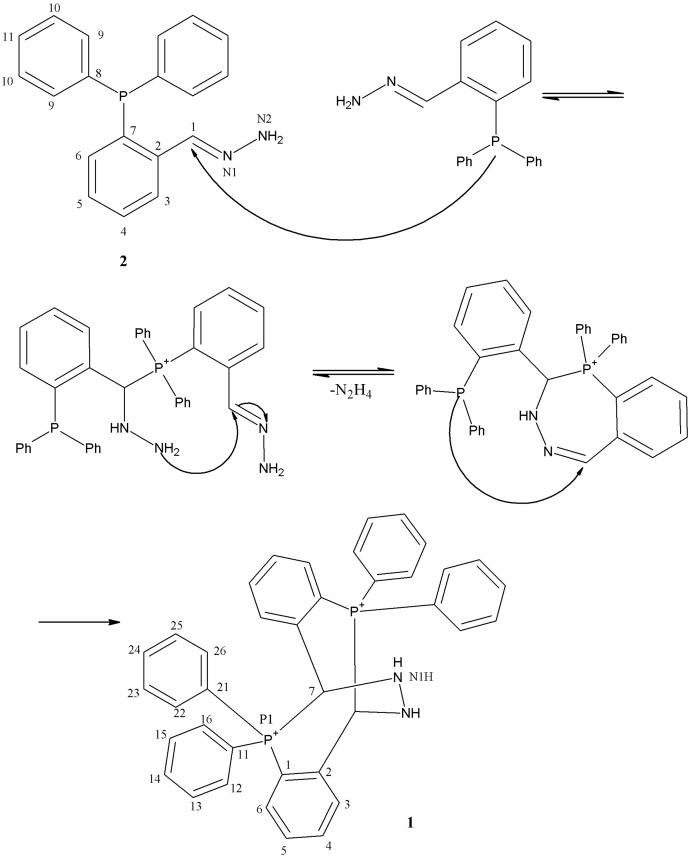
Mechanism of formation of **1**.

Since the presence of the electron-donating diphenylphosphino group in an aromatic aldehyde decreases the electrophilicity of the carbonyl carbon, and the electron withdrawing moiety decreases both the basicity and nucleophilicity of the nitrogen atom in malonic acid dihydrazide, the reaction of 2-(diphenylphosphino)benzaldehyde and malonic acid dihydrazide in molar ratio 2:1 was performed in methanol under more acidic conditions (pH 1.5–2), affording 5,5,11,11-tetraphenyl-5,6,11,12-tetrahydro-6,12-biiminodibenzo[*b,f*][1,5]diphosphocinium diperchlorate **1** (Scheme 1) in 14.5% yield.

In attempts to synthesize complexes of Cd(II) and Co(II) by template synthesis, starting from the corresponding perchlorate salts (Cd(ClO_4_)_2_·6H_2_O or Co(ClO_4_)_2_·6H_2_O), 2-(diphenylphosphino)benzaldehyde and malonic acid dihydrazide in molar ratio 2:2:1 in methanol at pH 1.5–2, the title compound (**1**) was obtained as the reaction product. In the presence of Co(II) ions in the reaction solution, the unhydrated crystals of **1** were obtained in trace yield. When Cd(II) ions were present in the reaction solution after recrystallization from acetonitrile the hydrated crystals of **1** were obtained in 16.6% yield, meaning that cadmium had no significant influence on the reaction. The purification procedure of **1** using column chromatography on silica gel was unsuccessful, the main product which was isolated being (*1E*)-[2-(diphenylphosphino)benzylidene]hydrazine **2** (Scheme 1) in 19.0% yield.

### 2.2. X-ray Crystallographic Analysis

**Figure 1:**
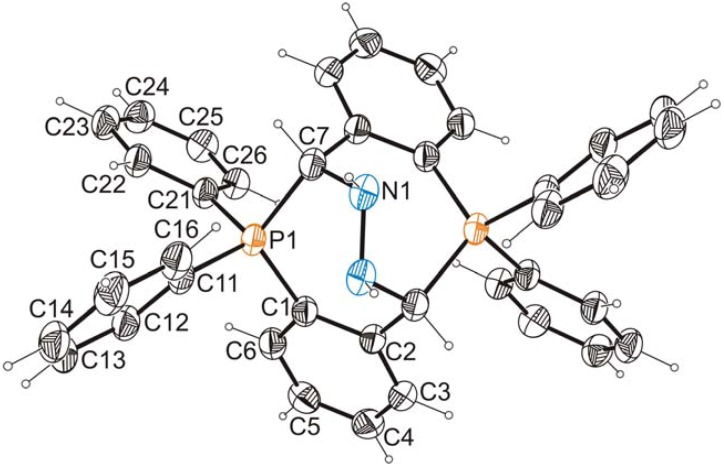
View of the diphosphoniumcation present in hydrated and non-hydrated crystals of **1**. Displacement ellipsoids are drawn at 30% probability level. As the molecular cation shown in this figure occupies two-fold symmetry site, the labels indicate only its symmetry independent part.

Both the anhydrous (compound **1**) and hydrated (compound **1** **×** **H_2_O**) crystals have been subjected to X-ray structure analysis. The results of these analyses are illustrated in Figure 1, which shows the dicationic moiety, the heteroanalogue of bicyclo[3.3.2]decane, common for both investigated crystal structures. Details of data collection and refinement and selected parameters describing the geometry of the molecular core are given in Tables S1 and S2 of the Supplementary Material.

The molecular cations of **1** formally possess two-fold symmetry and contain two stereogenic centers (C7 and its symmetry equivalent in Figure 1) that are both of the same configuration (*R* for the cation depicted in Figure 1). Hence, the molecules are chiral, but their crystals, being centrosymmetric, are not. Moreover, the molecular cation of **1** **×** **H_2_O** (shown in Figure 1) has an exact *C*_2_ symmetry in the solid state while that of **1** has only an approximate *C*_2_ symmetry (Figure S1, Supplementary Material). The conformation of the cation is nearly the same in the hydrated and the anhydrous crystals; both seven-membered rings adopt a distorted twist-chair conformation, while the eight-membered ring conformation is a twist-boat. This conformation leads to the dihedral angles of 82.07(9) in **1** and 82.37(10)° in **1** **×** **H_2_O** between the benzene rings fused with the eight-membered ring. The orientation of the N–N bridge is close to staggered, the H–N–N–H torsion angles being −34 and −31°, in **1** and **1** **×** **H_2_O**, respectively. The P···P distances are 3.9911(10) and 4.0136(14) Å, for **1** and **1** **×** **H_2_O**, respectively, and the intra-ring C–P–C angles are systematically wider: 115.38(14), 113.79(14) for **1** and 114.38(13)° for **1** **×** **H_2_O**, than the remaining valence angles on phosphorous, which are in the range from 103.79(14) to 114.32(16)°, the mean value being 108.3(3.5)°. Undoubtedly, the ring system seeks to impose planarity, which is also reflected in the intra-ring P–C^*^–N valence angles of 112.3(2), 113.2(2) and 112.1(2)°. The P–C bond lengths (mean P–C(sp^3^) 1.865(2) Å, mean P–C(sp^2^) 1.801(4) Å) show little deviation from the standard values listed in International Tables for X-ray crystallography [14].

Packing in crystals is mostly governed by ionic and hydrogen-bond interactions. Hydrogen-bond parameters are listed in Table S3, and hydrogen bonding scheme is illustrated in Figures S2 and S3, Supplementary Material. In **1**, the H-bonds are solely of the two-center NH···O type and are formed with the perchlorate groups as acceptors, while in the hydrated crystals the hydrogen bonds are also of the OH···O type. In **1** **×** **H_2_O** crystals the NH group (and its two-fold symmetry equivalent) forms three-center hydrogen bonds by donating its protons to both the perchlorate and water oxygen atoms, and water molecule acts as a donor to the perchlorate oxygen. While in the unhydrated crystals discrete supramolecular units formed by hydrogen bonded molecules around the inversion centers are observed (Figure S2), in **1** **×** **H_2_O** addition of water molecules enables extension of hydrogen bonded molecules into tapes running along the *c*-direction and consisting of fused rings formed by hydrogen-bonded molecules around the translation related inversion centers (Figure S3).

### 2.3. NMR Spectra

**Figure 2:**
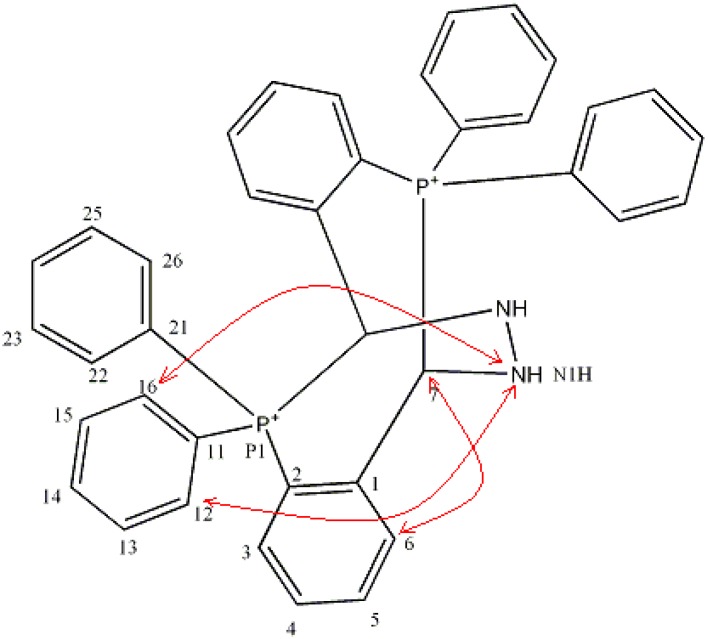
Important correlations obtained from NOESY spectrum.

The molecular cation of **1** possesses three types of NMR active nuclei: ^1^H, ^13^C and ^31^P. Crystals of **1** **×** **H_2_O** were dissolved in CD_3_CN and NMR spectra were recorded. Results obtained from 1D NMR spectra were not sufficient to resolve the structure, since signals in ^1^H-NMR spectrum are complex overlapping multiplets. Additional data were obtained from 2D NMR experiments: COSY, NOESY, HSQC and HMBC. There are no differences between the structures obtained by X-ray crystallography and NMR spectroscopy indicating the stability of **1** in CD_3_CN solution. Important correlations obtained from NOESY spectrum in support of this statement are shown on Figure 2.

### 2.4. Reaction Mechanism

Considering the mechanism of formation of **1**, we propose that malonic acid dihydrazide underwent a degradation to hydrazine under the experimental conditions, because of the coordination of the metal ion to the carbonyl oxygen lone pair due to its Lewis acidity. The metal ions of perchlorates are hard Lewis acids owing to the high delocalization of the negative charge in the perchlorate anion [15]. The liberated hydrazine reacted with 2-(diphenylphosphino)benzaldehyde, affording **1**. In order to confirm the assumption, a reaction of 2-(diphenylphosphino)benzaldehyde and hydrazine sulfate in methanol at pH 1.5−2 in the presence of perchloric acid was performed and **1** was obtained in 18.7% yield. The probable reaction mechanism for this condensation is shown in Scheme 1. This reaction involved aldehyde and hydrazine, which form hydrazone *in situ*, followed by addition of nucleophilic phosphorus, cyclization, elimination of hydrazine, and addition of the second phosphorous atom to the endocyclic hydrazone moiety to afford the final product. Although the literature describiing such reactions is scarce, evidence for both the nucleophilic addition of a phosphine to the carbon-nitrogen double bond [16] and the transhydrazonation [17] exist in the literature.

There are very few structural analogues of **1**. The most similar one is an eight-membered cyclic compound without a bridge, 5,6,11,12-tetrahydro-5,5,11,11-tetraphenyldibenzo[*b,f*][1,5]diphosphocinium dibromide, which was obtained by the intermolecular cyclization reaction of two molecules of [2-(bromomethyl)phenyl]diphenylphosphine [18].

### 2.5. Biological Activity

The biological activity of **1** and **2** was tested by the brine shrimp test (toxicity to *A. salina*). The results of this test can be extrapolated to cell-line toxicity and anti-tumor activity [19,20]. The brine shrimp lethality test showed a good activity for **1** with, LC_50_ 52.7 μM, and a moderate activity for **2** with LC_50_ 3.82 mM.

**Figure 3:**
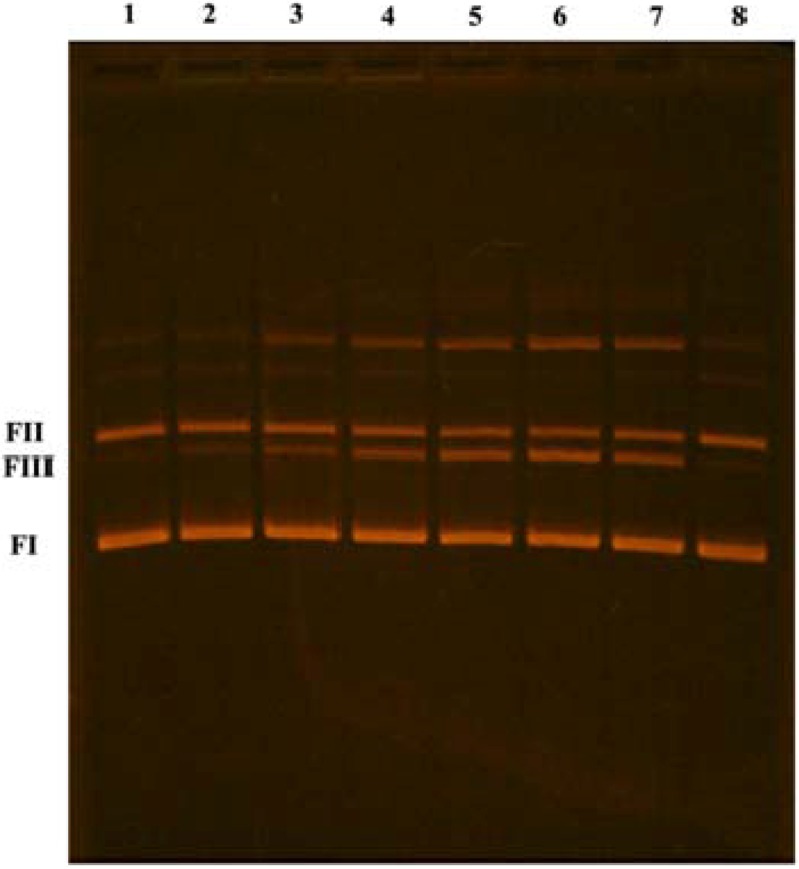
Agarose gel electrophoresis of plasmid pUC18 DNA.pUC18 (213 ng) without (lane 1) and pUC18 (213 ng) with 5 nmol, 10 nmol, 15 nmol, 20 nmol, 25 nmol and 30 nmol of **1** (lanes 2, 3, 4, 5, 6 and 7 respectively), pUC18 (213 ng) with acetonitrile (6μL) (lane 8).

The ability of **1** to cleave double-stranded plasmid DNA was investigated using an agarose gel electrophoresis assay. This assay allows assessment of DNA strand cleavage by measuring the conversion of uncut supercoiled (form I) plasmid DNA to the nicked form (form II) and linear form (form III). The electrophoretic separations are shown in Figure 3. Compound **1** relaxes the supercoiled plasmid DNA (form I) and nicked form (form II) to the linear form (form III) in a dose-dependent manner. Another form, with the lowest mobility, was noticed, and its amount also increased with the increase of concentration of **1**. The DNA cleaving activity of **1** is probably a consequence of various structural features: its positive charge would enable it to engage in electrostatic interactions with DNA, its 8-membered macrocyclic moiety could enable groove binding to DNA, and its P, N acetal is the reactive center which can covalently modify DNA.

## 3. Experimental

### 3.1. General

2-(Diphenylphosphino)benzaldehyde (97%), and malonic acid dihydrazide (97%) were obtained from Sigma-Aldrich (Saint Louis, MO, USA), hydrazine sulfate (99%) was obtained from Merck (Darmstadt, Germany). All solvents (methanol, acetonitrile) and chemicals used for DNA studies were reagent grade and used without further purification. All buffer solutions for investigations of interactions of compounds with DNA were prepared in deionized sterile water and filtered through 0.2 µm filters (Nalgene, Lima Ohio, OH, USA). Ethidium bromide and plasmid pUC18 were purchased from Serva (Heidelberg, Germany). IR spectra were recorded on a Perkin-Elmer FT-IR 1725X spectrometer using the ATR technique in the region 4,000–400 cm^−1^. ^1^H-NMR (200 MHz) and ^13^C-NMR (50 MHz) for **2** were recorded on a Varian-Gemini 2000 spectrometer in CDCl_3_ using TMS as internal standard for ^1^H and ^13^C. ^1^H-NMR (500 MHz), ^13^C-NMR (125 MHz) and 2D NMR spectra for **1** were recorded on a Bruker Avance 500 spectrometer in CD_3_CN using TMS as internal standard for ^1^H and ^13^C. All spectra were measured at 25 °C. Compound **2** was characterized on the basis of NMR spectroscopy results: 1D (^1^H, ^13^C, DEPT). Compound **1** was characterized on the basis of NMR spectroscopy results: 1D (^1^H, ^13^C, DEPT), 2D (COSY, NOESY) as well as 2D ^1^H–^13^C heteronuclear correlation spectra (HSQC, HMBC). ^31^P NMR (202 MHz) spectrum for **1** was recorded on a Bruker Avance 500 spectrometer using 85% phosphoric acid as external standard. Mass spectra were taken on a 6210 TOF LC/MS coupled with an Agilent Technologies 1200 Series HPLC system. A Submarine Mini-gel Electrophoresis Unit (Hoeffer HE 33) with an EPS 300 power supply was used for electrophoresis experiments. The stained gels were illuminated under a Vilber-Lourmat UV transilluminator (Marne-la-Vallée, France) at 312 nm and photographed with a Panasonic DMC-LZ5 Lumix Digital Camera through a DEEP YELLOW 15 (Tiffen, NY, USA) filter.

### 3.2. Synthesis of 5,5,11,11-Tetraphenyl-5,6,11,12-tetrahydro-6,12-biiminodibenzo[b,f][1,5]diphosphociniumDiperchlorate (**1**) from Malonic Acid Dihydrazide and 2-(Diphenylphosphino)benzaldehyde

To a solution containing 0.03 g (0.23 mmol) malonic acid dihydrazide and 0.15 g (0.52 mmol) 2-(diphenylphosphino)benzaldehyde in methanol (15 mL) four drops of conc. HClO_4_ were added and the pH value was adjusted to 1.5−2. After stirring the mixture for six hours at 42 °C, a white precipitate formed was filtered off and washed with cold methanol. Single crystals of **1** were obtained by vapor diffusion using acetonitrile, pH of which was adjusted to 4.6 with CH_3_COOH. Yield 14.5% (29 mg), mp 242 °C. ^1^H-NMR (500 MHz, CD_3_CN) δ 6.47 (2H, m, N1H), 6.75 (2H, m, C6H), 7.18 (6H, m, C14H, C24H, C7H), 7.63 (2H, complex signal, C4H), 7.71 (2H, m, C5H), 7.76 (4H, m, C22H, C26H), 7.86 (4H, m, C12H, C16H), 7.92 (10H, m, C3H, C13H, C15H, C23H, C25H); ^13^C-NMR (125 MHz, CD_3_CN) δ 73.5 (d, *^1^J* = 58.9 Hz, C7), 117.8 (d, *^2^J* = 3.12 Hz, C2, partly masked by the signal of the solvent), 119.6 (d, *^1^J* =7 9.0 Hz, C21), 122.4 (d, *^1^J* = 92.9 Hz, C11), 130.2 (s, C4), 131.4 (m, C22, C26), 131.7 (m, C12, C16, C23, C25), 133.6 (m, C6), 135.2 (s, C5), 135.9 (m, C13, C15), 137.3 (m, C14, C24), 139.7 (m, C3), 142.0 (m, C1); ^31^P-NMR (CD_3_CN) δ 28.21; IR (vs-very strong, s-strong, m-medium, w-weak): 3644 (w), 2584 (w), 3537 (w), 3298 (s), 2361 (w), 1621 (w), 1584 (w), 1479 (w), 1438 (m), 1096 (vs), 752 (m), 687 (m), 620 (m), 537 (w), 505 (m) cm^−1^; MS (ESI) *m/z* Calcd for C_38_H_32_N_2_P_2_ 578.2034, found 578.2024. Elemental analysis calcd for C_38_H_34_N_2_P_2_Cl_2_O_9_: N 3.52%, C 57.37%, H 4.31%, found: N 3.52%, C 57.39%, H 4.35%.

### 3.3. Synthesis of 5,5,11,11-Tetraphenyl-5,6,11,12-tetrahydro-6,12-biiminodibenzo[b,f][1,5]diphosphocinium Diperchlorate (**1**) from Malonic Acid Dihydrazide and 2-(Diphenylphosphino)benzaldehyde in the Presence of Cd(ClO_4_)_2_·6H_2_O

Cd(ClO_4_)_2_·6H_2_O 0.11 g (0.26 mmol), malonic acid dihydrazide 0.03 g (0.23 mmol) and 2-(diphenylphosphino)benzaldehyde 0.15 g, (0.52 mmol) were dissolved in methanol (15 mL). Four drops of HClO_4_ were added and the mixture was stirred for six hours at 42 °C. A white precipitate formed was filtered off and washed with cold methanol. Single crystals of **1** were obtained by vapor diffusion using acetonitrile, pH of which was adjusted to 4.6 with CH_3_COOH. Yield 16.6% (33 mg). The white crude precipitate which was obtained from this experimental procedure was purified by column chromatography through silica gel, eluting with a gradient of 10% to 50% acetonitrile in toluene to afford (*1E*)-[2-(diphenylphosphino)benzylidene]hydrazine **2** as a yellow solid. Yield 19.0% (30 mg), mp 288 °C. ^1^H-NMR (200 MHz, CDCl_3_) δ 7.06−7.28 (3H, m, C4H, C11H), 7.31−7.64 (12H, m, C5H, C6H, C9H, C10H, N2H), 8.25−8.31 (1H, m, C3H), 9.22 (1H, s, C1H); ^13^C-NMR (50 MHz, CDCl_3_) δ 159.2 (d, *^3^J* = 6.4 Hz, C1), 137.6 (d, *^1^J* = 6.4 Hz, C8), 133.0 (d, *^2^J* = 11.9 Hz, C6), 132.6 (s, C7), 131.8 (s, C11), 131.4 (d, *^2^J* = 10.0 Hz, C9), 130.5 (s, C2), 129.6 (d, *^3^J* = 12.8 Hz, C5), 128.3 (s, C3), 128.3 (d, *^3^J* = 12.8 Hz, C10), 127.7 (s, C4); IR (vs-very strong, s-strong, m-medium, w-weak): 3446 (m), 2365 (w), 2254 (w), 1436 (w), 1183 (m), 1109 (m), 1054 (vs), 1028 (vs), 824 (w), 761 (m), 723 (w), 692 (w), 543 (m) cm^−1^. MS (ESI) *m/z* Calcd for C_19_H_17_N_2_P 304.11295, found 304.11369. Elemental analysis calcd for C_19_H_17_N_2_P: C 74.99%, N 9.21%, H 5.63%, found: C 74.78%, N 9.13%, H 5.75%.

### 3.4. Synthesis of 5,5,11,11-Tetraphenyl-5,6,11,12-tetrahydro-6,12-biiminodibenzo[b,f][1,5]diphosphocinium Diperchlorate (**1**) from Malonic Acid Dihydrazide and 2-(Diphenylphosphino)benzaldehyde in the Presence of Co(ClO_4_)_2_·6 H_2_O

Co(ClO_4_)_2_·6H_2_O 0.09 g (0.25 mmol), malonic acid dihydrazide 0.03 g (0.23 mmol) and 2-(diphenylphosphino)benzaldehyde 0.15 g (0.52 mmol) were dissolved in methanol (10 mL). Several drops of HClO_4_ were added and the mixture was stirred for 30 min at 53 °C. The brownish-red precipitate arose from solution with the white single crystals of **1**. Yield in traces.

### 3.5. Synthesis of 5,5,11,11-Tetraphenyl-5,6,11,12-tetrahydro-6,12-biiminodibenzo[b,f][1,5]diphosphocinium Diperchlorate (1) from Hydrazine Sulfate and 2-(Diphenylphosphino)Benzaldehyde

To a solution containing 0.03 g (0.23 mmol) hydrazine sulfate and 0.15 g (0.52 mmol) 2-(diphenylphosphino)benzaldehyde in methanol (15 mL) four drops of conc. HClO_4_ were added and the pH value was adjusted to 1.5−2. After stirring the mixture for three hours at 42 °C, a white precipitate was filtered off and washed with cold methanol. Single crystals of **1** were obtained by vapor diffusion using acetonitrile, pH of which was adjusted to 4.6 with CH_3_COOH. Yield 18.7% (38 mg).

### 3.6. X-ray Single Crystal Measurements

CCDC 862205 and 862206 contain the supplementary crystallographic data for **1** and **1** **×** **H_2_O**. These data can be obtained free of charge via http://www.ccdc.cam.ac.uk/conts/retrieving.html, or from the Cambridge Crystallographic Data Centre, 12 Union Road, Cambridge CB2 1EZ, UK; fax: (+44)1223-336-033; or e-mail: deposit@ccdc.cam.ac.uk.

The crystals of **1** and **1** **×** **H_2_O** were mounted in a loop containing some amount of perfluoroether as protector and flash cooled on SuperNova kappa-geometry diffractometer to 130(2) K. The temperature of the samples was controlled with an Oxford Instruments Cryosystem cold-nitrogen-gas blower. The diffractometer was equipped with fine-focus CuKα radiation (λ = 1.5405 Å). The intensity data were corrected for absorption effects [21]. The structures were solved by direct methods using SHELXS-86 [22] and refined by least-squares techniques with SHELXL-97 [23]. Anisotropic displacement parameters were employed for non-hydrogen atoms. For tertiary and aromatic CH groups the positions of the hydrogen atoms were calculated at standardized distances of 1.00 and 0.95 Å, respectively. The positions of the NH hydrogens and a hydrogen atom being part of the C_2_ symmetrical water molecule were determined from the difference Fourier maps but their bond distances were standardized to the values of 0.92 and 0.85 Å, respectively. All H-atoms were refined using a riding model with isotropic temperature factors 20% higher than the isotropic equivalent for the atom to which the H-atom was bonded. At the final stages of the structure refinement it appeared evident that the crystal of **1** contains structural voids each containing the residual electron density of about 0.7 eÅ^−3^. We have assumed that the residual density in the voids represents atmospheric gases and used the SQUEEZE [24] program to account for the presence of this electron density. Hence, the atom list does not contain the gas atoms captured in the crystal. The SQUEEZE program indicated the presence of 8 voids each of the volume ca. 34Å^3^ containing 4 electrons. The presence of voids would explain lower density of **1** compared to its hydrated crystal form (Table S1). Siemens [25] computer graphics program was used to prepare drawings. The relevant crystal data collection and refinement parameters are listed in Table S1 and selected bond distances and angles are reported in Table S2 of the Supplementary Material.

### 3.7. The Brine Shrimp Test

A teaspoon of lyophilized eggs of the brine shrimp *Artemia salina* was added to 0.5 L of the artificial sea water containing several drops of yeast suspension (3 mg of dry yeast in 5 mL distilled water), and air was passed through the suspension thermostated at 18–20 °C, under illumination for 48 h. Hatched nauplii were used in further experiments.

The tested substances were dissolved in appropriate solvents (**1** in acetonitrile, **2** in chloroform) and then in various amounts applied to paper discs 2 cm in diameter. Paper discs were placed on the bottom of the glass vial into which was added 5 mL of artificial sea water, 1–2 drops of yeast suspension, and about 15–20 hatched nauplii.

The vials were left at room temperature under illumination for 24 h, and afterwards live and dead nauplii were counted. LC_50_ was defined as the concentration of substances that causes death of 50% nauplii.

### 3.8. DNA Cleavage Experiment

Plasmid pUC18 was prepared by transformation of a clone from *Escherichia coli* RR1 (pUC18, 2686 bp, purchased from Sigma, Saint Louis, MO, USA) into electrocompetent *E. coli* DH5α strain cells according to the protocol for growing *E. coli* culture overnight in LB medium at 37 °C by electroporation with the “Gene Pulser” (Bio-Rad, USA) [26]. The plasmid DNA from *E. coli* clones was isolated by modified method of alkali lysis [27] and purified with the “JetStar” kit (Genomed, Aventura, USA) using anion-exchange column. After final washing step with ice-cold 70% ethanol, DNA pellet was air-dried and finally resuspended in 150 μL sterile H_2_O and stored at −20 °C. The concentration of plasmid DNA (213 ng/μL of pUC18) was determined by measuring the absorbance of the DNA-containing solution at 260 nm. One optical unit corresponds to 50 μg/mL of double stranded DNA.

Stock solution (5 mM) of **1** was prepared in acetonitrile. Plasmid DNA was incubated for 4 min at 95 °C, followed by incubation at 4 °C for the next 20 min. Then 213 ng of pUC18 in a 20 μL reaction mixture in 20 mM Tris 20 mM NaCl buffer pH 7.92, were incubated with different concentrations (0.25 mM, 0.50 mM, 0.75 mM, 1.00 mM, 1.25 mM and 1.5 mM) of **1** at 37 °C, for 2 h. The reaction mixtures were vortexed from time to time. The reaction was terminated by short centrifugation at 10,000 rpm and adding 7 μL of loading buffer (0.25% bromophenol blue, 0.25% xylene cyanol FF and 30% glycerol in TAE buffer, pH 8.24 (40 mM Tris-acetate, 1 mM EDTA). The samples were subjected to electrophoresis on 1% agarose gel (GE Healthcare Life sciences, Uppsala, Sweden) prepared in TAE buffer pH 8.24. The electrophoresis was performed at a constant voltage (80 V) for about 1.5 h (until bromophenol blue had passed through 75% of the gel). After electrophoresis, the gel was stained for 30 min by soaking it in an aqueous ethidium bromide solution (0.5 μg/mL), and after that was visualized under UV light.

## 4. Conclusions

Reported here is the preparation of a racemic diphosphonium compound that contains two nearly perpendicular benzene rings fused with an eight-membered ring and a hydrazine bridge. This compound, for which a variety of derivatives with potential biological activity could be synthesized, might be of use due to the fact that chiral molecules that lock two aromatic rings in almost perpendicular planes are of special interest as DNA probes.

## Supplementary Materials

Crystal data (Figure S1 and Table S1), selected geometrical parameters (Table S2), hydrogen-bond parameters (Table S3, Figures S2 and S3) and spectral data (Figures S4-S17). Detailed information can be accessed at: http://www.mdpi.com/1420-3049/17/3/2567/s1.
