# Supplementary material for: Synthesis, Spectral and Solid State Characterization of a New Bioactive Hydrazine Bridged Cyclic Diphosphonium Compound
Source: Molecules. 2012 Mar 2;17(3):2567–78. doi: 10.3390/molecules17032567 (PMC6268038; doi:10.3390/molecules17032567)

Supplementary Material

Synthesis, Spectral and Solid State Characterization of a New Bioactive Hydrazine Bridged Cyclic Diphosphonium Compound

Milica Milenković [1], Beata Warżajtis [2], Urszula Rychlewska [2], Dušanka Radanović [3], Katarina Anđelković [1], Tatjana Božić [1], Miroslava Vujčić [3] and Dušan Sladić [1,*]

[1] Faculty of Chemistry, University of Belgrade, Studentski trg 12-16, 11000 Belgrade, Serbia
[2] Faculty of Chemistry, A. Mickiewicz University, Grunwaldzka 6, 60-780 Poznań, Poland
[3] Institute of Chemistry, Technology and Metallurgy, University of Belgrade, Njegoševa 12, P.O. Box 815, 11000 Belgrade, Serbia

* Author to whom correspondence should be addressed; E-Mail: dsladic@chem.bg.ac.rs; Tel.: +38111-333-66-79; Fax: +38111-263-60-61.

List of contents:

Crystal data: Figure S1 and Table S1
Selected geometrical parameters: Table S2
Hydrogen-bond parameters: Table S3, Figure S2 and Figure S3
NMR spectra of compound 1: Figure S4-S11
HRMS spectrum of compound 1: Figure S12
IR spectrum of compound 1: Figure S13
NMR spectra of compound 2: Figure S14-S16
IR spectrum of compound 2: Figure S17

Figure S1. View of the molecular cation of **1** present in the anhydrous crystals. Displacement ellipsoids are drawn at 40% probability level.

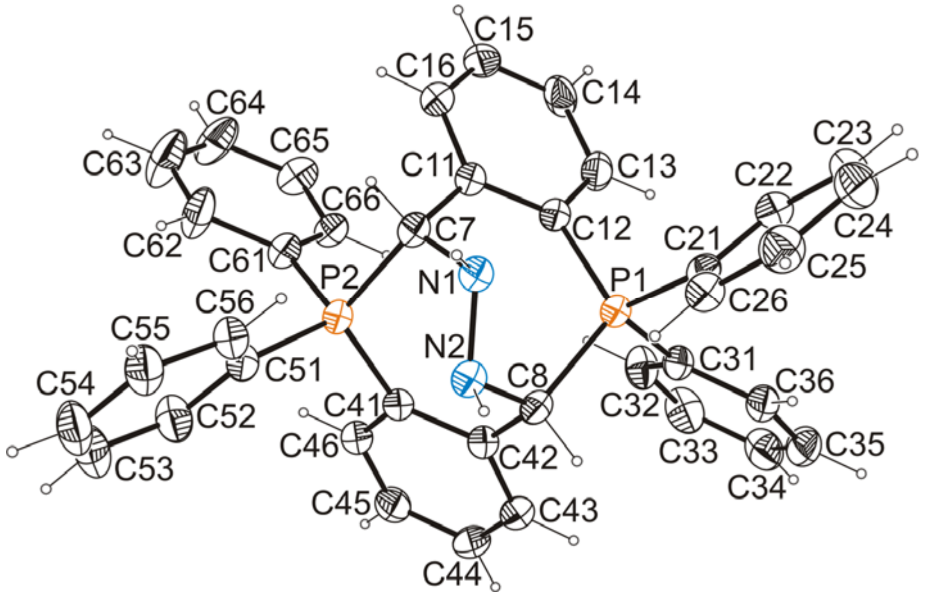

Figure S2. Hydrogen bonding scheme in the crystal structure of **1**.

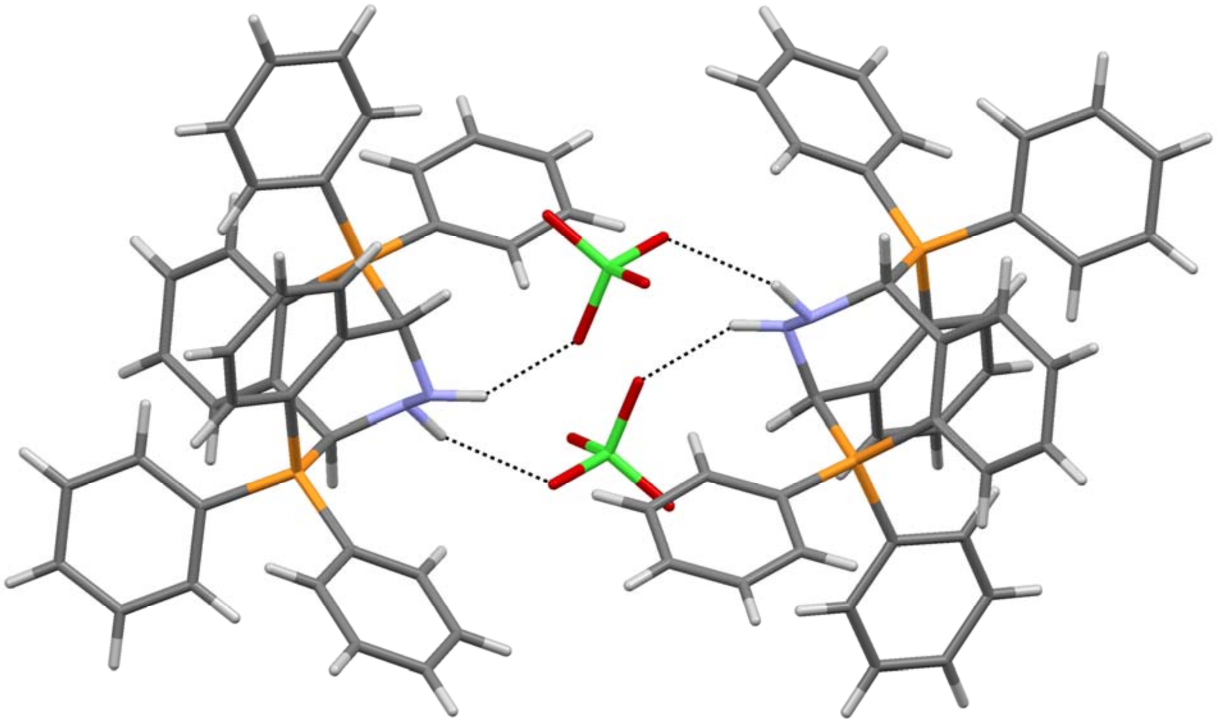

Figure S3. Hydrogen bonding scheme in the crystal structure of **$1xH_2O$**.

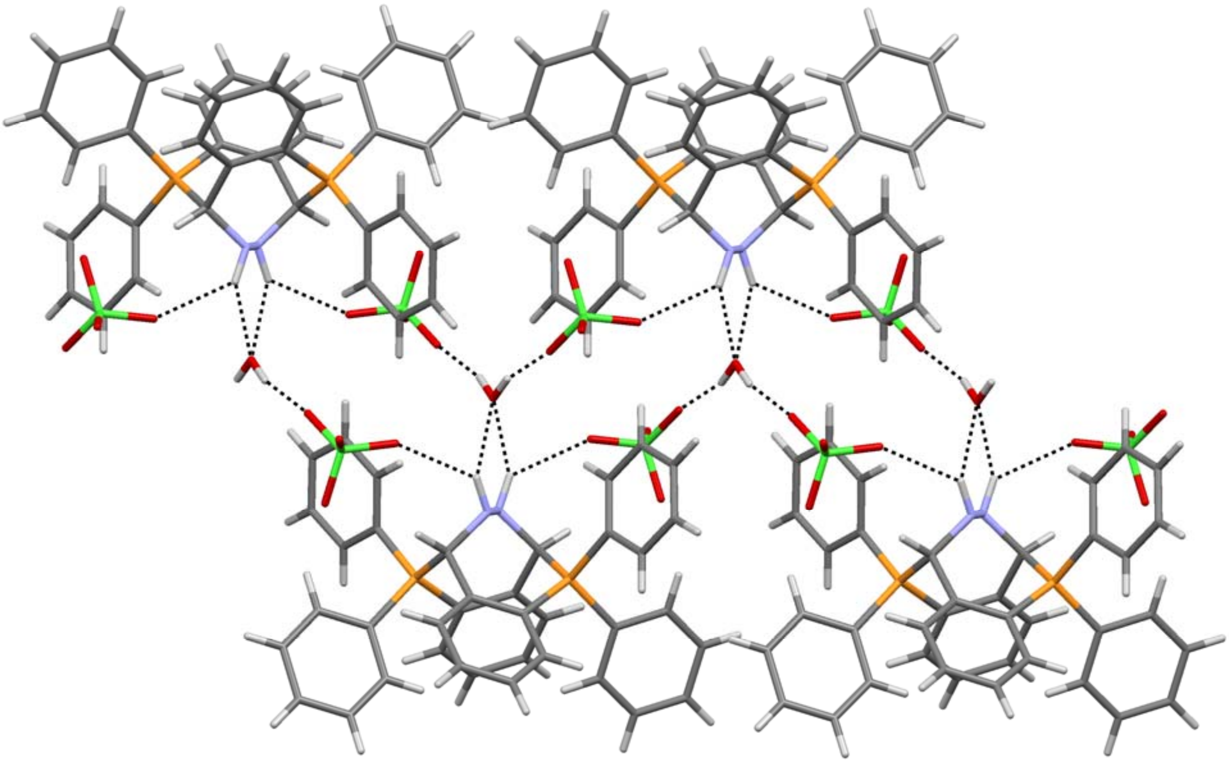

Table S1. Experimental details.

	**1**	**1 x H_2O**
Crystal data		
Chemical formula	$C_{38}H_{32}N_2P_2\cdot 2(ClO_4)$	$C_{38}H_{32}N_2P_2\cdot 2(ClO_4)\cdot H_2O$
M_r	777.50	795.51
Crystal system, space group	Orthorhombic, *Pbca*	Monoclinic, *C*2/*c*
a, *b*, *c* (Å)	17.9459 (8), 15.9361 (5), 24.9474 (7)	18.3160 (12), 18.9967 (7), 12.3020 (8)
α, β, γ (°)	90, 90, 90	90, 123.050 (9), 90
V (Å^3)	7134.6 (4)	3587.8 (4)
Z	8	4
μ (mm^{-1})	2.97	2.98
Crystal size (mm)	0.20 × 0.05 × 0.02	0.30 × 0.15 × 0.03
Data collection		
T_{min}, T_{max}	0.896, 1.000	0.455, 1.000
No. of measured, independent and observed [$I > 2\sigma(I)$] reflections	25578, 7401, 4576	7694, 3169, 2674
R_{int}	0.071	0.024
Refinement		
$R[F^2 > 2\sigma(F^2)]$, $wR(F^2)$, S	0.057, 0.140, 0.93	0.059, 0.169, 1.07
No. of reflections	7401	3169
No. of parameters	469	240
$\Delta\rho_{max}$, $\Delta\rho_{min}$ (e Å^{-3})	0.54, −0.39	0.47, −0.61

Table S2. Selected geometric parameters (Å,°) *.

**1**		**1 x H2O**	
Bond lenghts			
N1—N2	1.450 (4)	N1—N1^{i}	1.450 (5)
N1—C7	1.450 (4)	N1—C7	1.459 (4)
N2—C8	1.456 (4)	*N1—C7*	*1.459 (4)*
P1—C8	1.866 (3)	P1—C7	1.862 (3)
P1—C12	1.805 (3)	P1—C1	1.802 (3)
P1—C31	1.806 (3)	P1—C21	1.800 (3)
P1—C21	1.797 (3)	P1—C11	1.793 (3)
P2—C7	1.866 (3)	*P1—C7*	*1.862 (3)*
P2—C41	1.808 (3)	*P1—C1*	*1.802 (3)*
P2—C61	1.799 (4)	*P1—C21*	*1.800 (3)*
P2—C51	1.796 (3)	*P1—C11*	*1.793 (3)*
Valence angles			
N2—N1—C7	109.0 (3)	N1^{i}—N1—C7	109.2 (2)
N1—N2—C8	108.8 (3)	*N1^{i}—N1—C7*	*109.2 (2)*
C12—P1—C8	113.79 (14)	C1—P1—C7	114.38 (13)
C12—P1—C21	109.88 (15)	C1—P1—C11	111.58 (14)
C12—P1—C31	107.59 (16)	C1—P1—C21	107.72 (14)
C21—P1—C31	106.58 (15)	C11—P1—C21	105.72 (13)
C21—P1—C8	114.32 (16)	C11—P1—C7	112.83 (14)
C31—P1—C8	104.04 (15)	C21—P1—C7	103.79 (14)
C41—P2—C7	115.38 (14)	*C1—P1—C7*	*114.38 (13)*
C41—P2—C51	113.09 (15)	*C1—P1—C11*	*111.58 (14)*
C41—P2—C61	105.35 (17)	*C1—P1—C21*	*107.72 (14)*
C51—P2—C61	106.51 (16)	*C11—P1—C21*	*105.72 (13)*
C51—P2—C7	111.73 (17)	*C11—P1—C7*	*112.83 (14)*
C61—P2—C7	103.69 (16)	*C21—P1—C7*	*103.79 (14)*

* Values written in italic style are generated by symmetry.

Table S2. *Cont.*

Torsion angles					
**1**				**1 x H_2O**	
Seven-membered rings					
N1—N2—C8—P1	8.6 (3)	N2—N1—C7—P2	10.0 (3)	N1^{i}—N1—C7—P1	9.5 (3)
N2—C8—P1—C12	−61.1 (3)	N1—C7—P2—C41	−60.6 (3)	N1—C7—P1—C1	−61.0 (2)
C8—P1—C12—C11	38.3 (3)	C7—P2—C41—C42	33.1 (3)	C2—C1—P1—C7	35.2 (3)
P1—C12—C11—C7	−10.4 (5)	P2—C41—C42—C8	−4.4 (4)	P1—C1—C2—C7^{i}	−5.6 (4)
C12—C11—C7—N1	37.7 (4)	C41—C42—C8—N2	34.8 (4)	C1—C2—C7^{i}—N1^{i}	33.9 (4)
C11—C7—N1—N2	−113.8 (3)	C42—C8—N2—N1	−114.9 (3)	C2—C7^{i}—N1^{i}—N1	−113.0 (3)
C7—N1—N2—C8	86.5 (3)	*C7—N1—N2—C8*	*86.5 (3)*	C7^{i}—N1^{i}—N1—C7	87.1 (4)
Eight-membered rings					
C7—C11—C12—P1	−10.4 (5)	C8—C42—C41—P2	−4.4 (4)	C7^{i}—C2—C1—P1	−5.6 (4)
C11—C12—P1—C8	38.3 (3)	C42—C41—P2—C7	33.1 (3)	C2—C1—P1—C7	35.2 (3)
C12—P1—C8—C42	62.1 (2)	C41—P2—C7—C11	63.7 (3)	C1—P1—C7—C2^{i}	61.6 (2)
P1—C8—C42—C41	−90.5 (3)	P2—C7—C11—C12	−87.2 (3)	P1—C7—C2^{i}—C1^{i}	−90.1(3)
Triphenylphosphine fragment					
C8—P1—C12—C11	38.3(3)	C7—P2—C41—C42	33.1(3)	C7—P1—C1—C2	35.2(3)
C8—P1—C21—C22	−13.0(3)	C7—P2—C51—C56	−3.5(3)	C7—P1—C11—C16	-10.1(3)
C8—P1—C31—C32	82.8(3)	C7—P2—C61—C66	78.6(3)	C7—P1—C21—C26	84.74(3)

Symmetry code(s): (i) −*x*, *y*, −*z* + 1/2. * Values written in italic style are generated by symmetry.

Table S3. Hydrogen-bond parameters.

	D–H (Å)	D…A (Å)	H…A (Å)	D-H…A (°)	Symmetry operations on A
**1**					
N1–H1A···O3	0.92	2.966(4)	2.19	142	1.5-x, -0.5+y, z
N2–H2···O1	0.92	2.959(5)	2.15	146	0.5+x, 0.5-y, 1-z
**1 x H_2O**					
N1–H1···O1W	0.92	2.944(4)	2.23	134	
N1–H1···O1	0.92	3.231(3)	2.54	132	-0.5+x, 0.5-y, -0.5+z
O1W–H1O···O4	0.85	3.238(3)	2.42	162	-0.5+x, 0.5+y, -1+z

Figure S4. ^{1}H NMR (500 MHz) spectrum of compound **1**.

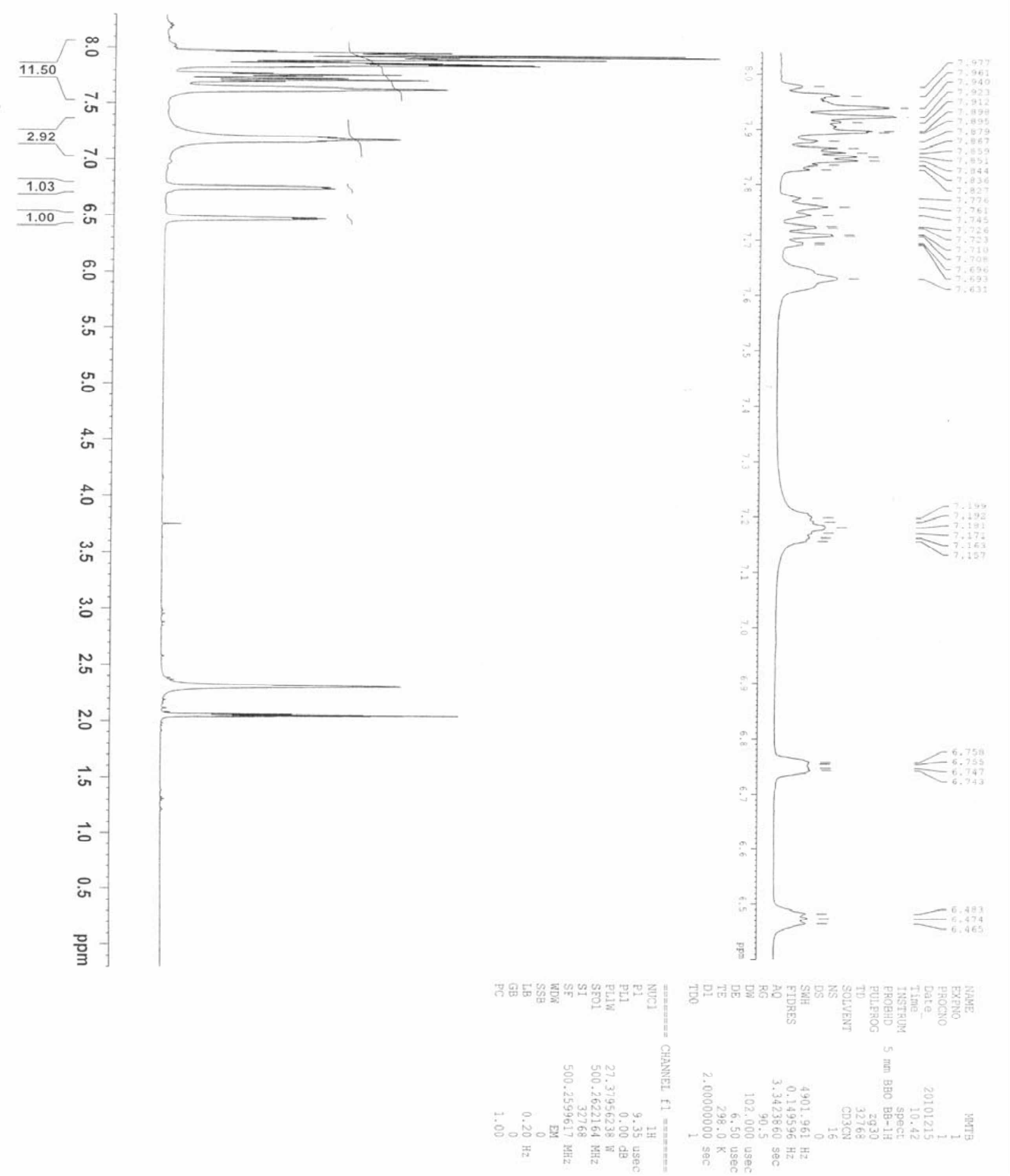

Figure S5. ^{13}C NMR (125 MHz) spectrum of compound **1**.

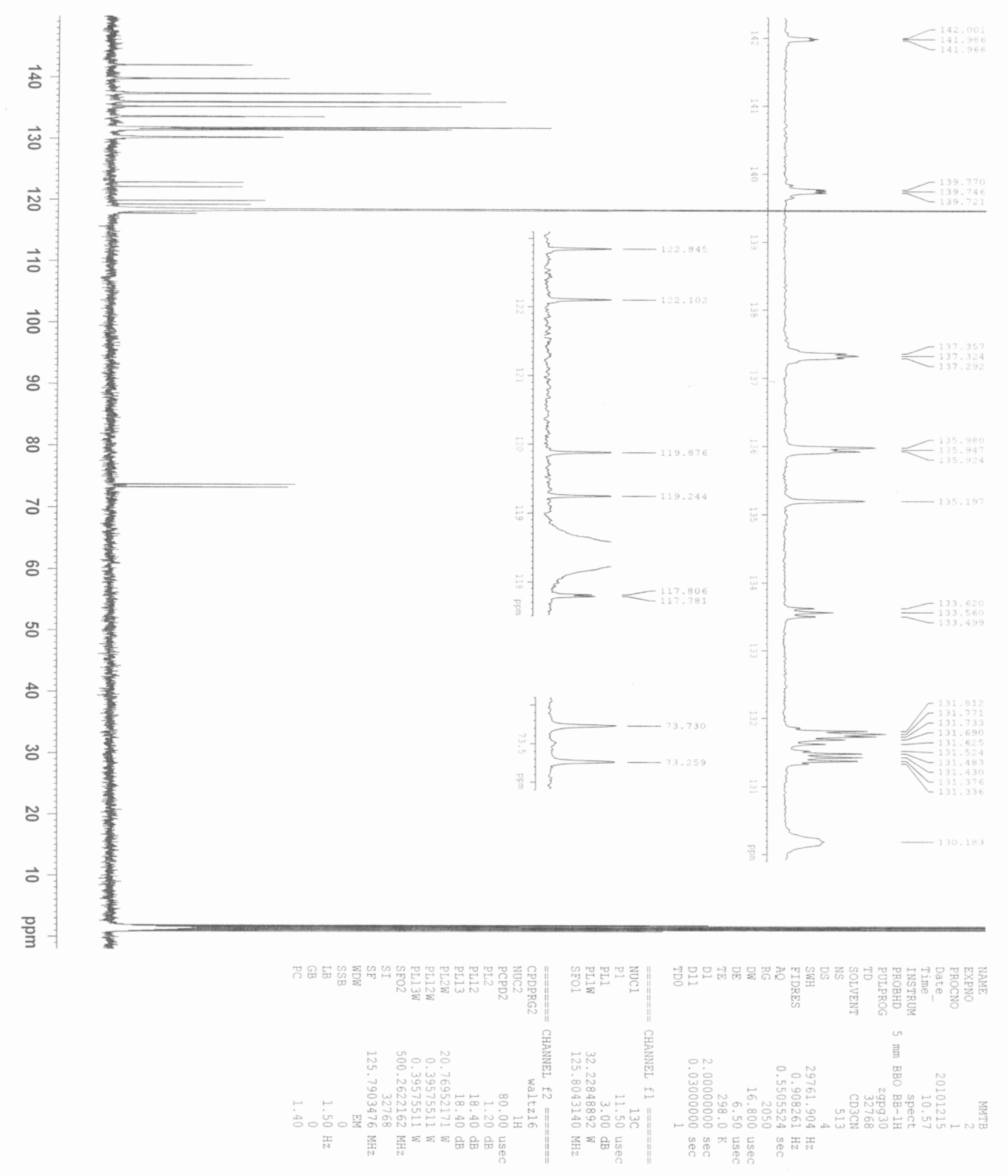

Figure S6. DEPT spectrum of compound **1**.

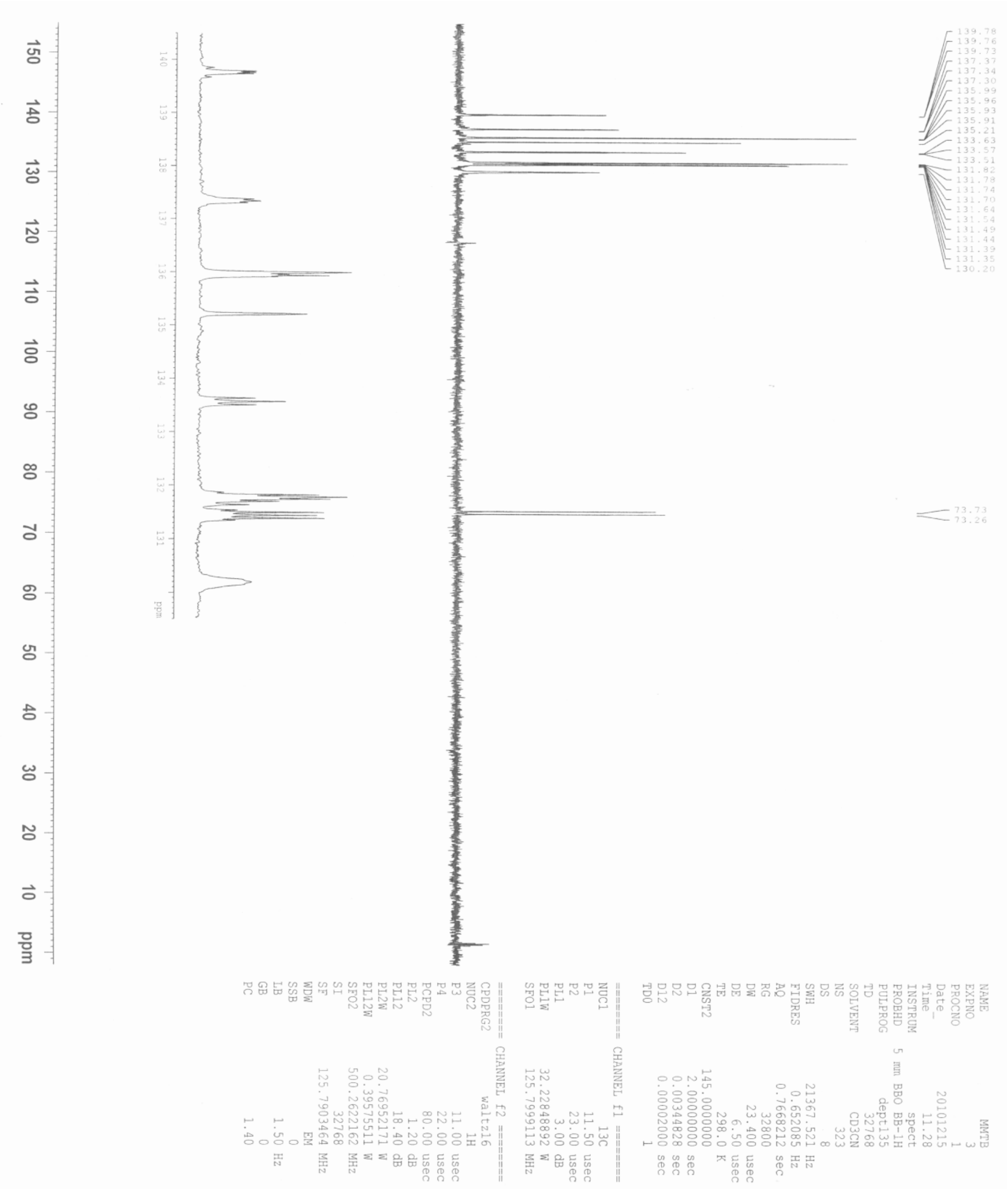

Figure S7. COSY spectrum of compound **1**.

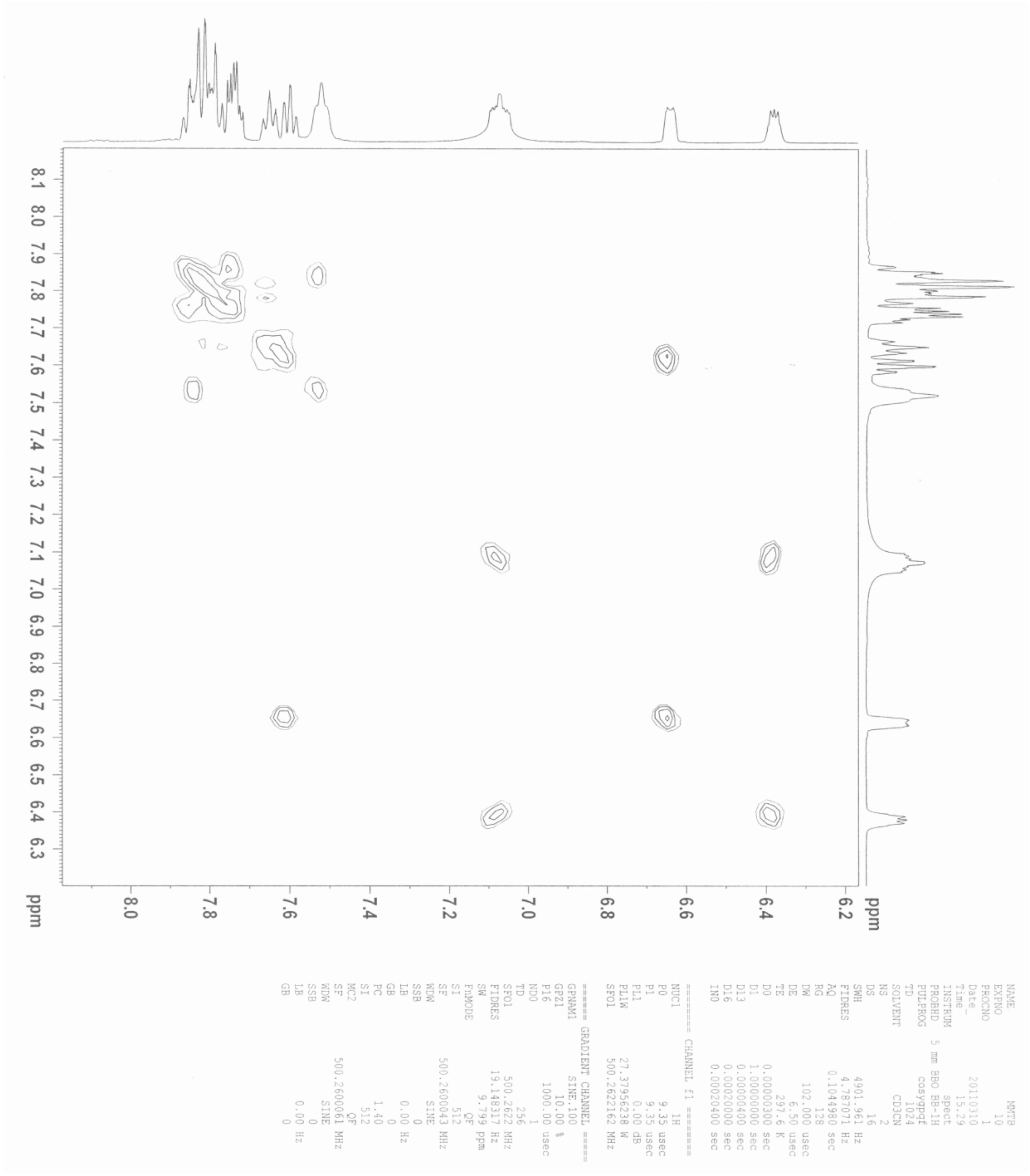

Figure S8. NOESY spectrum of compound **1**.

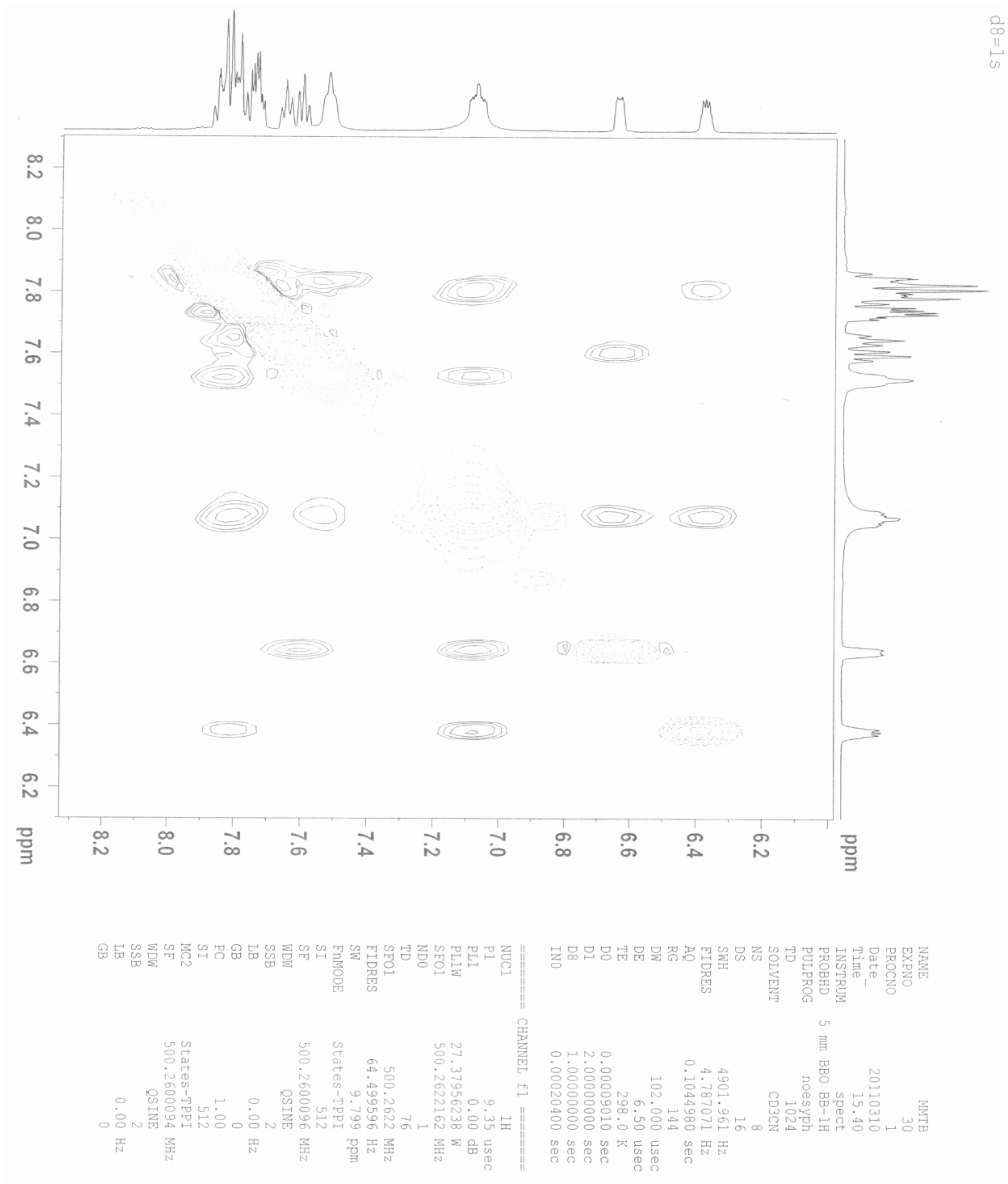

Figure S9. HSQC spectrum of compound **1**.

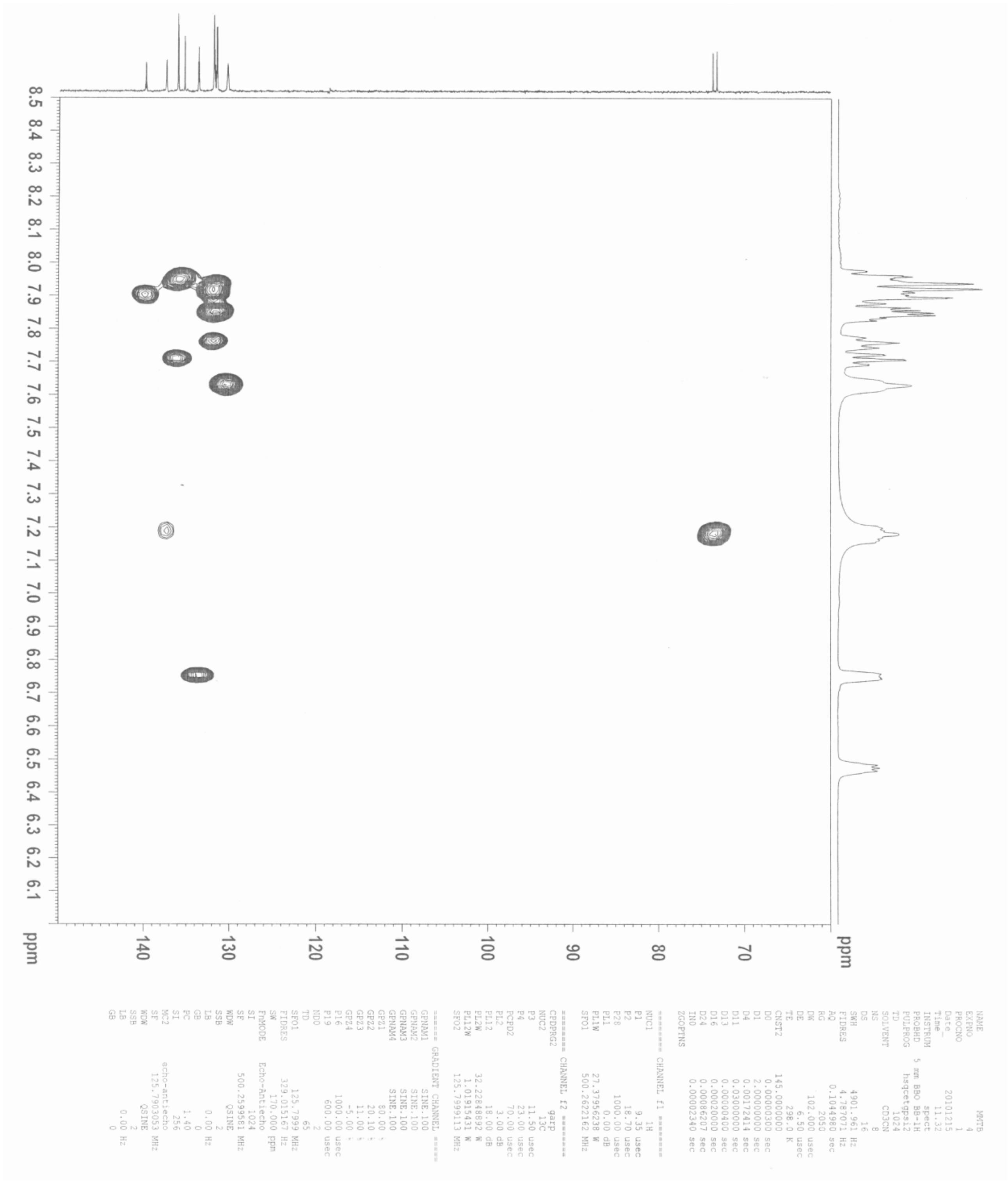

Figure S10a. HMBC spectrum of compound **1**.

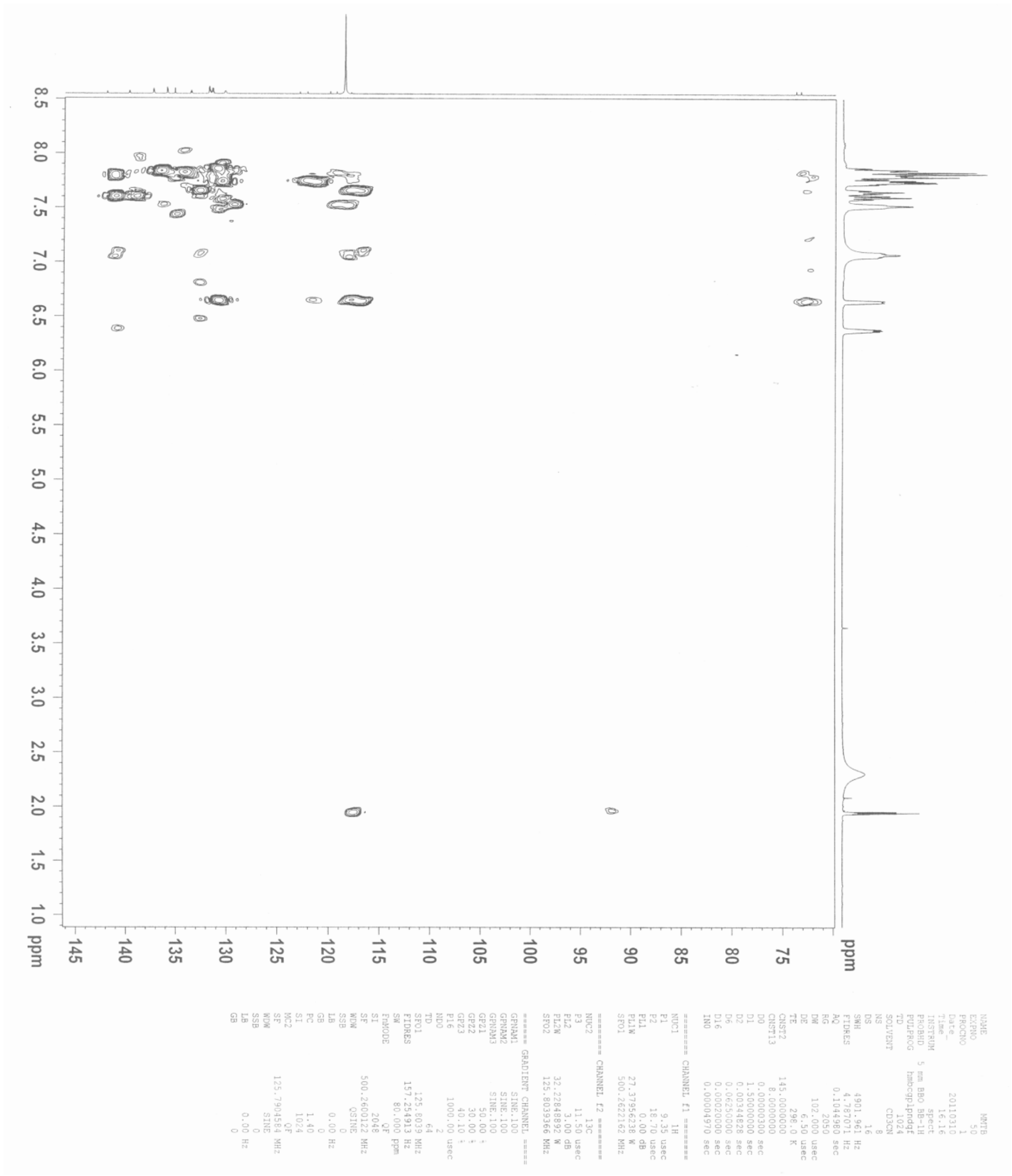

Figure S10b. HMBC spectrum of compound **1**.

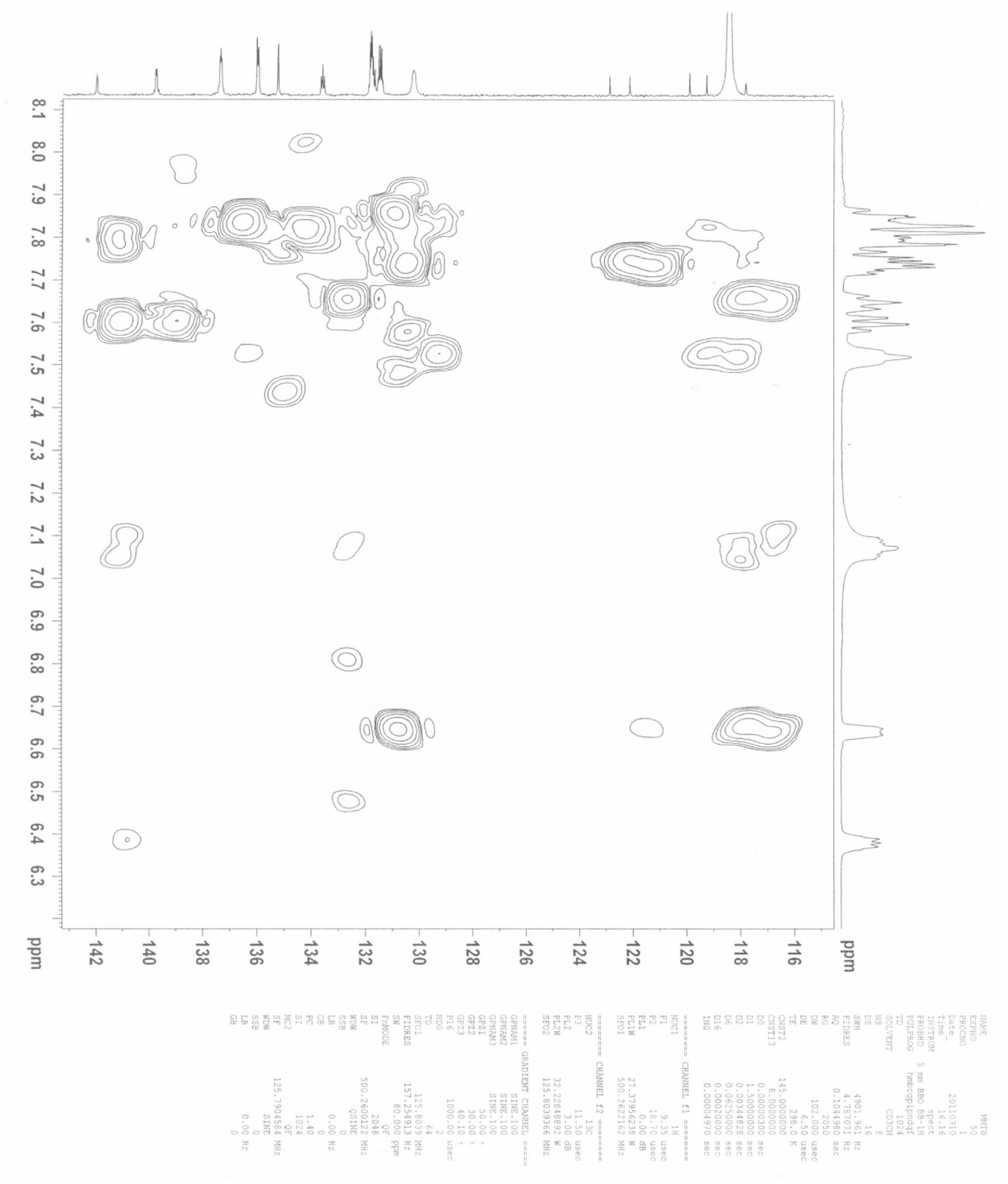

Figure S11. ^{31}P spectrum of compound **1**.

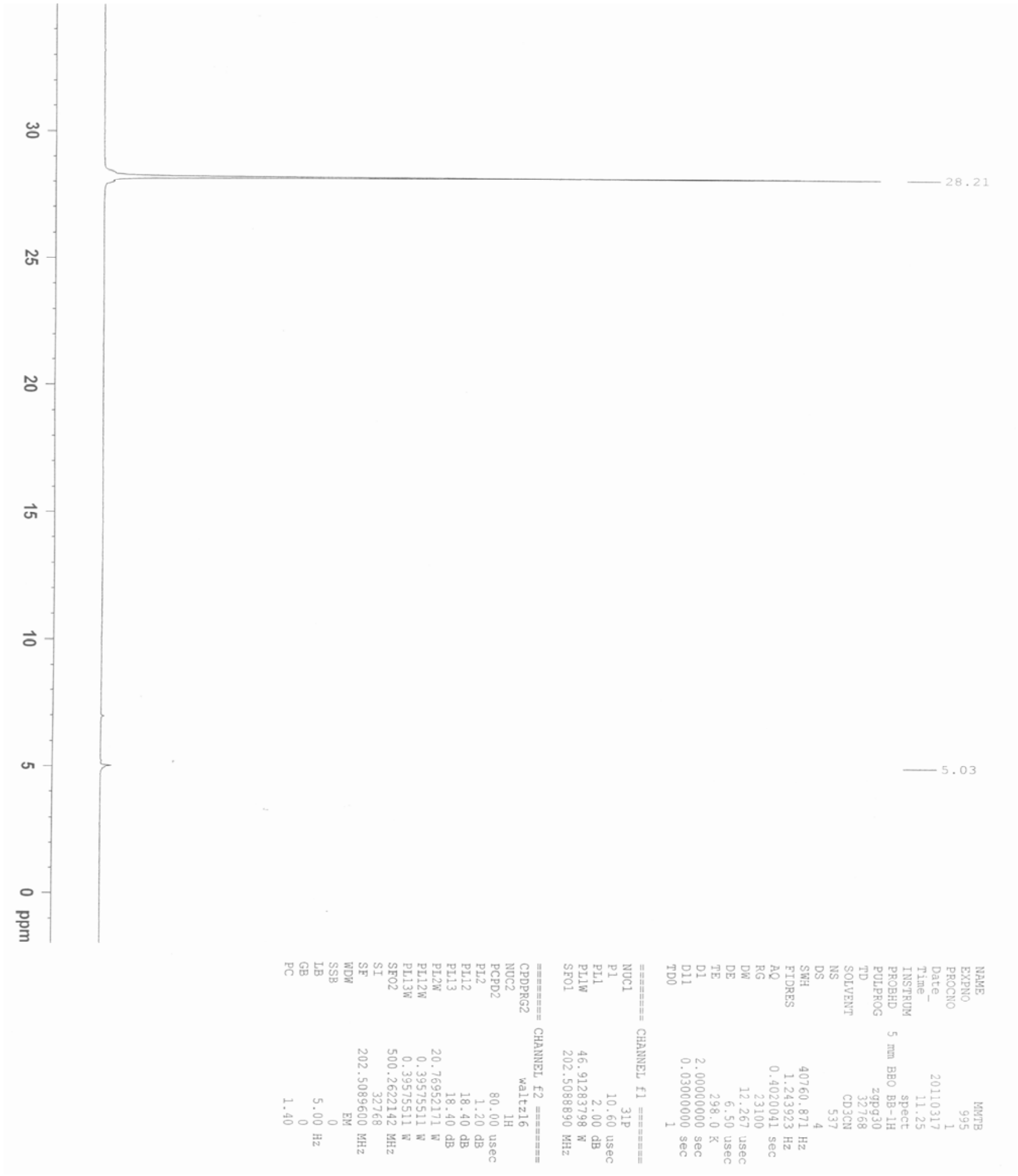

Figure S12. HRMS spectrum of compound **1**.

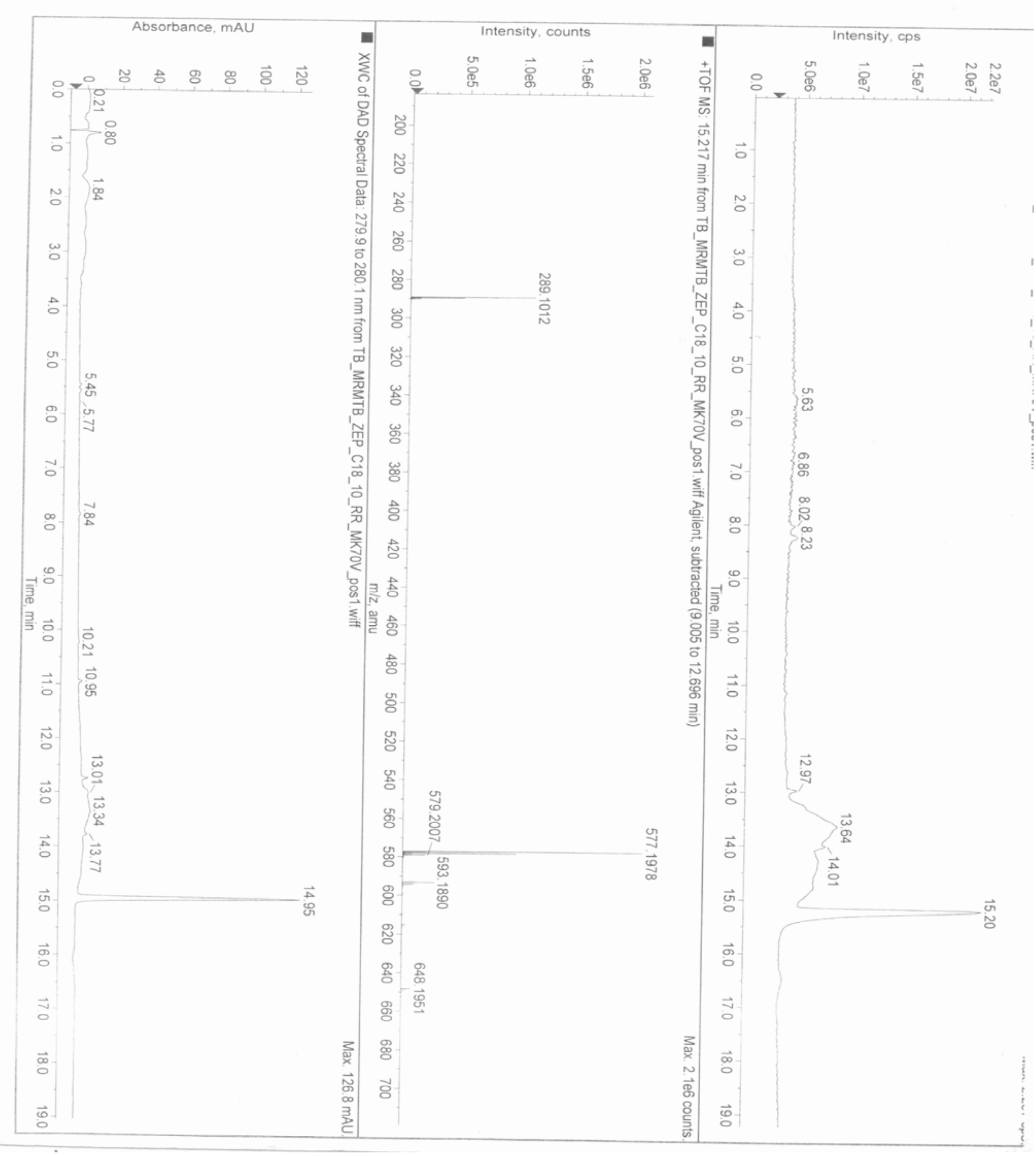

Figure S13. IR spectrum of compound **1**.

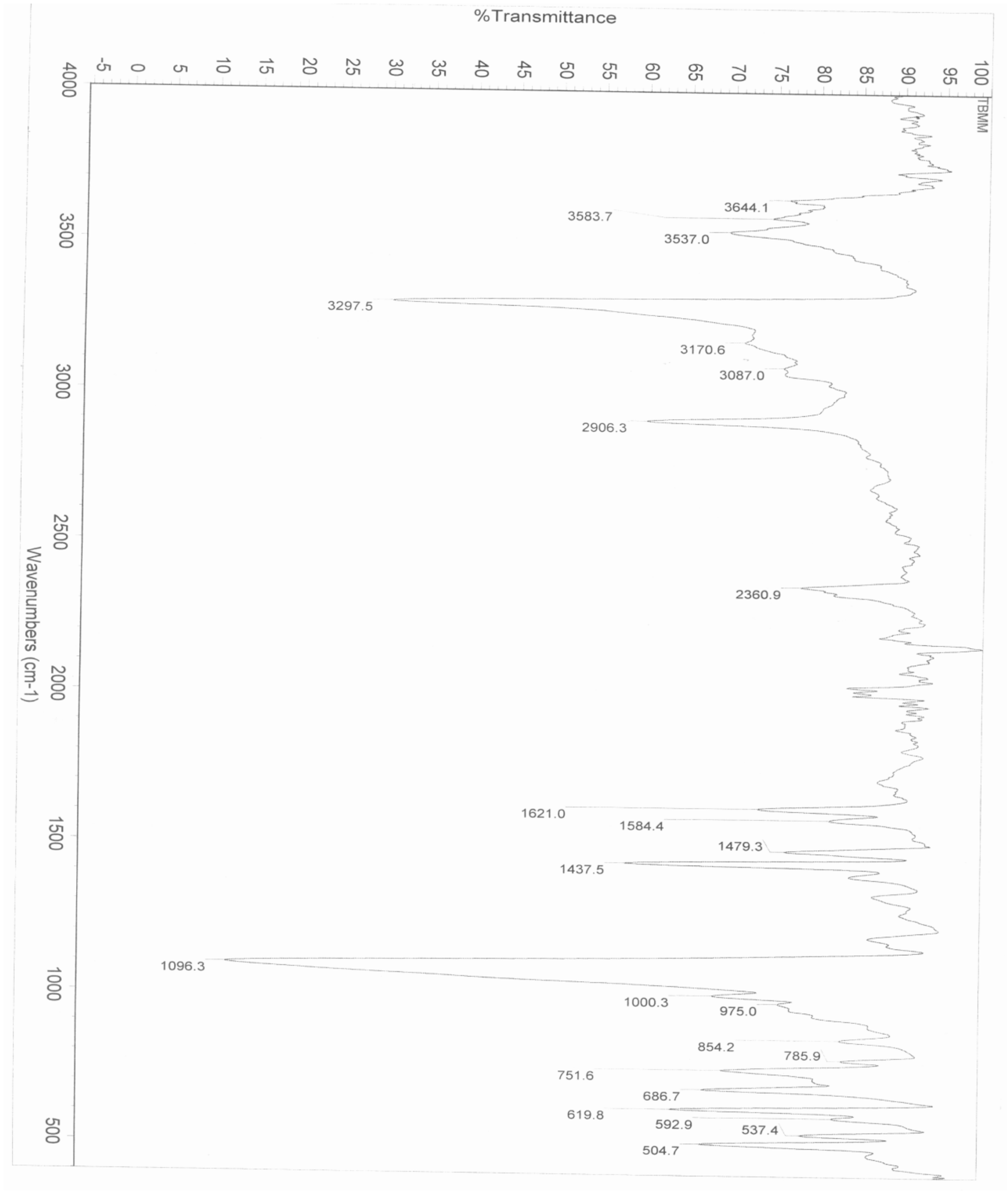

Figure S14a. ^{1}H-NMR (200 MHz) spectrum of compound **2**.

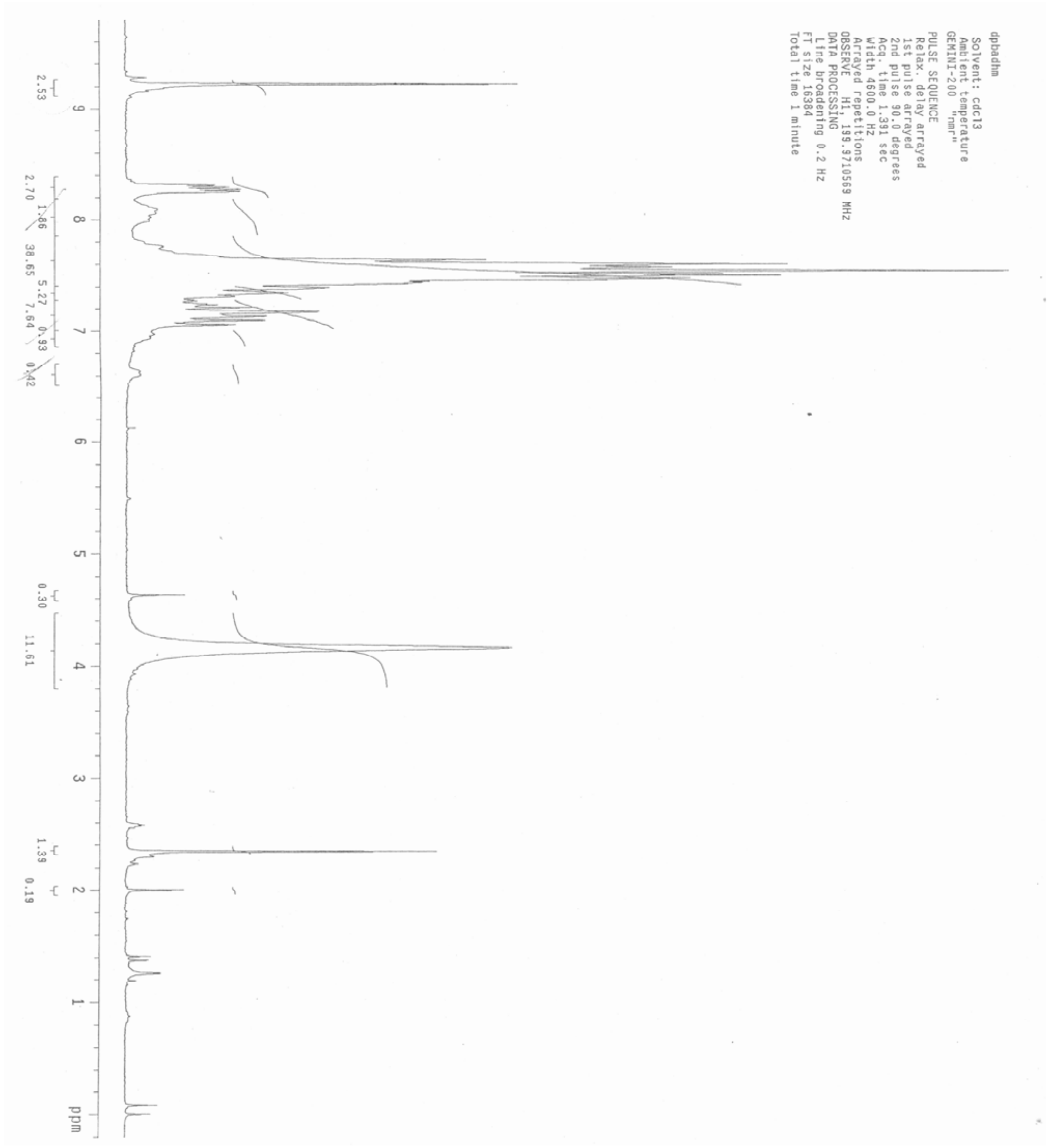

Figure S14b. ^{1}H-NMR (200 MHz) spectrum of compound **2**.

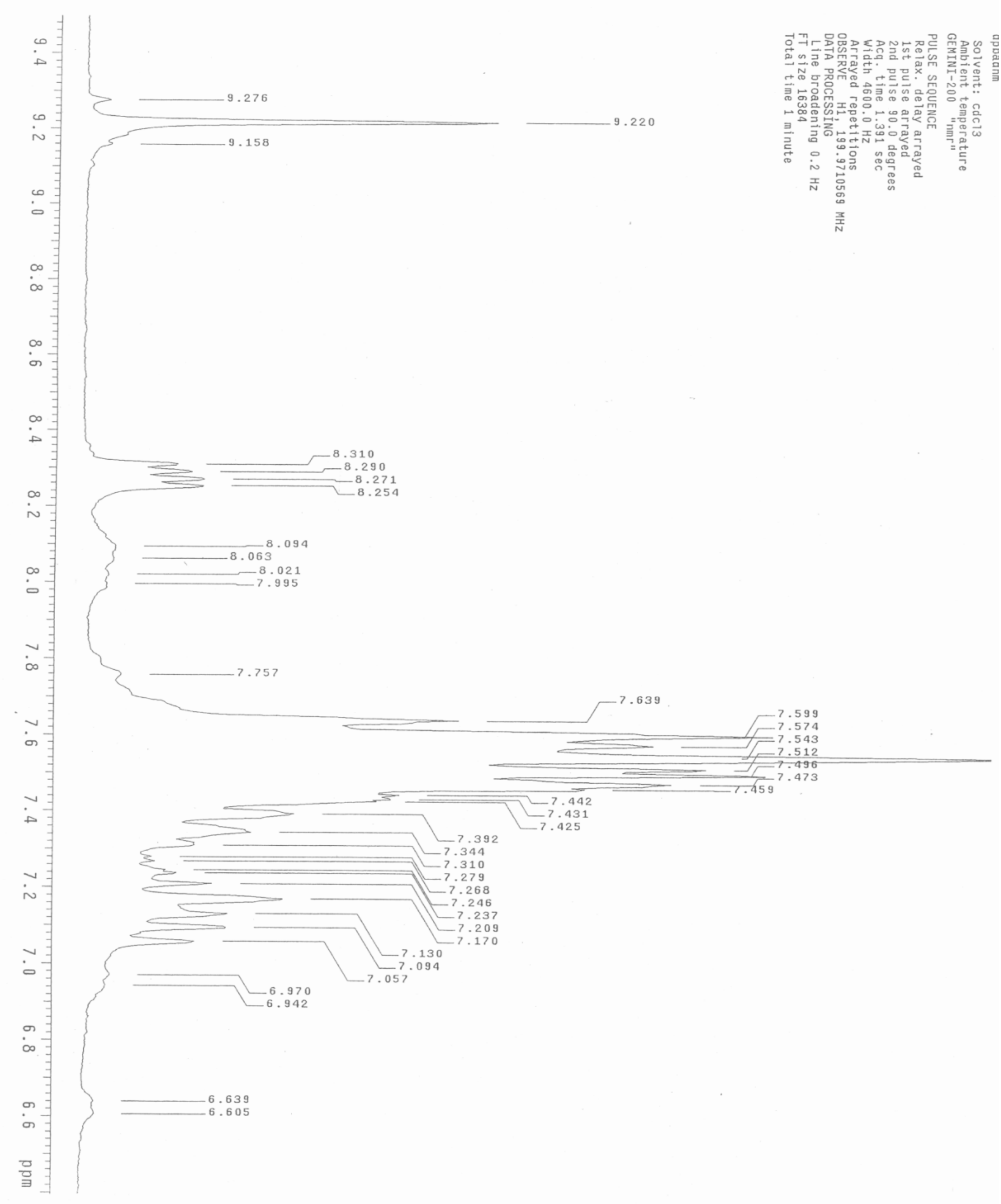

Figure S15a. ^{13}C-NMR (50 MHz) spectrum of compound **2**.

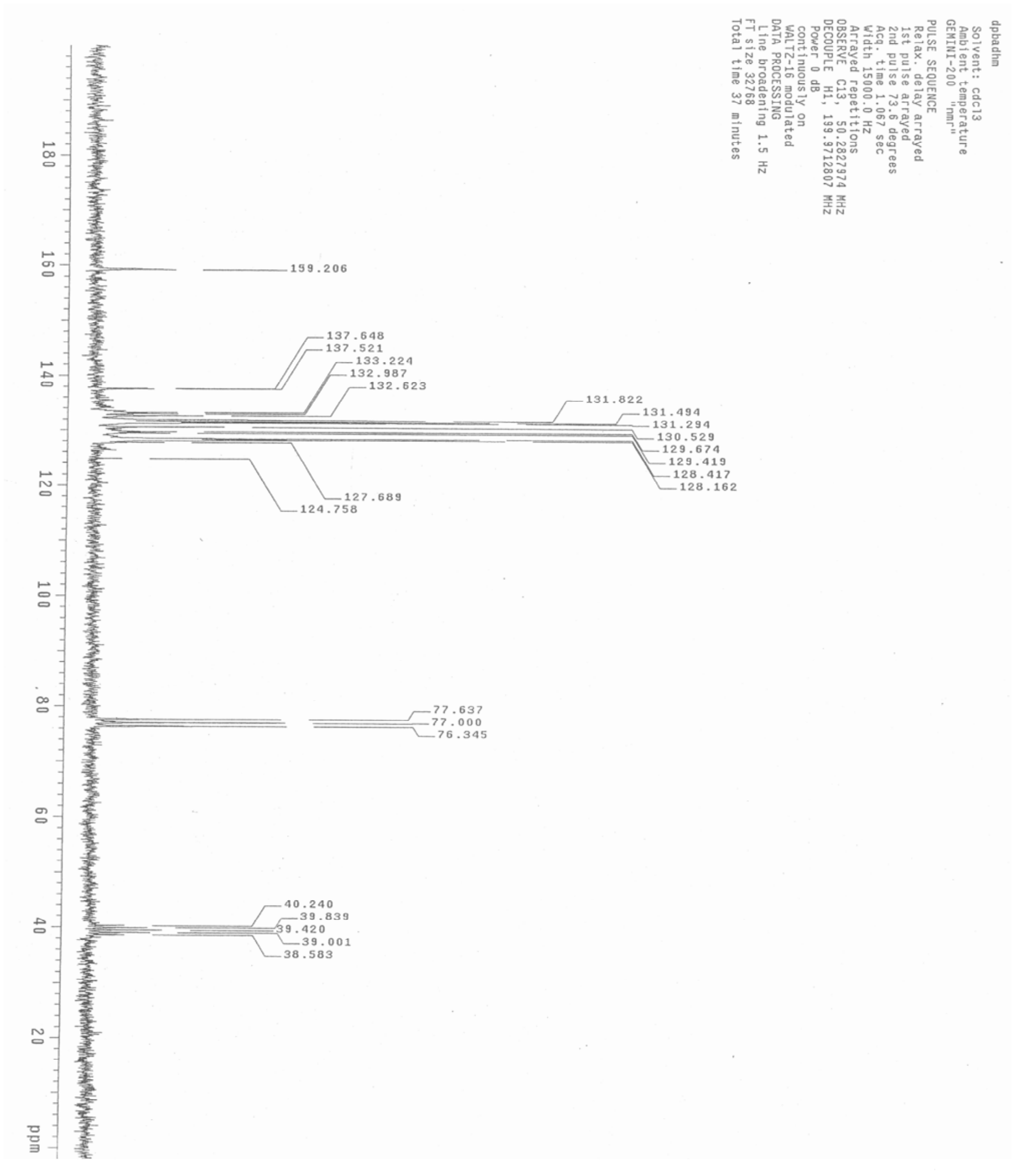

Figure S15b. ^{13}C-NMR (50 MHz) spectrum of compound **2**.

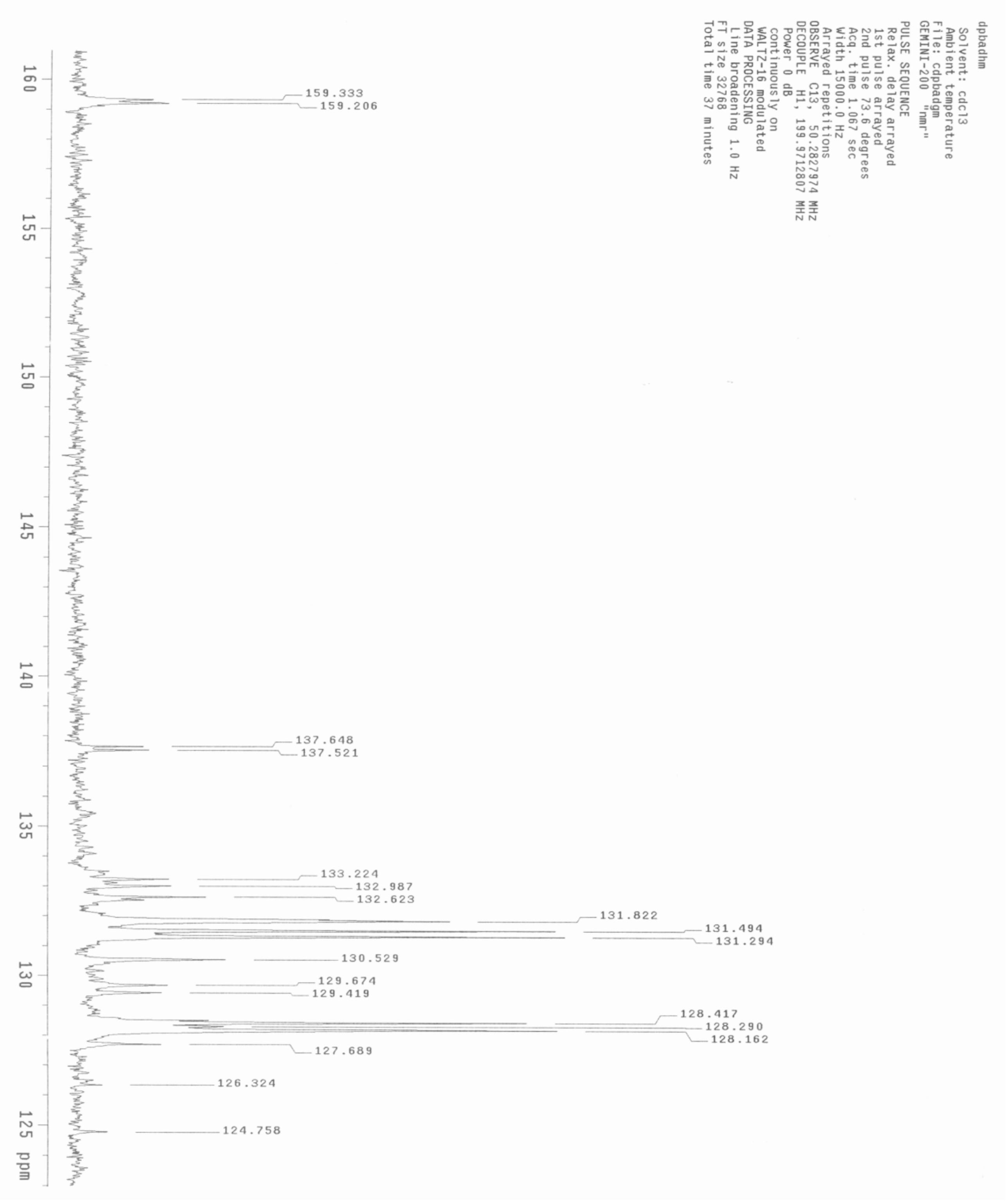

Figure S16. DEPT spectrum of compound **2**.

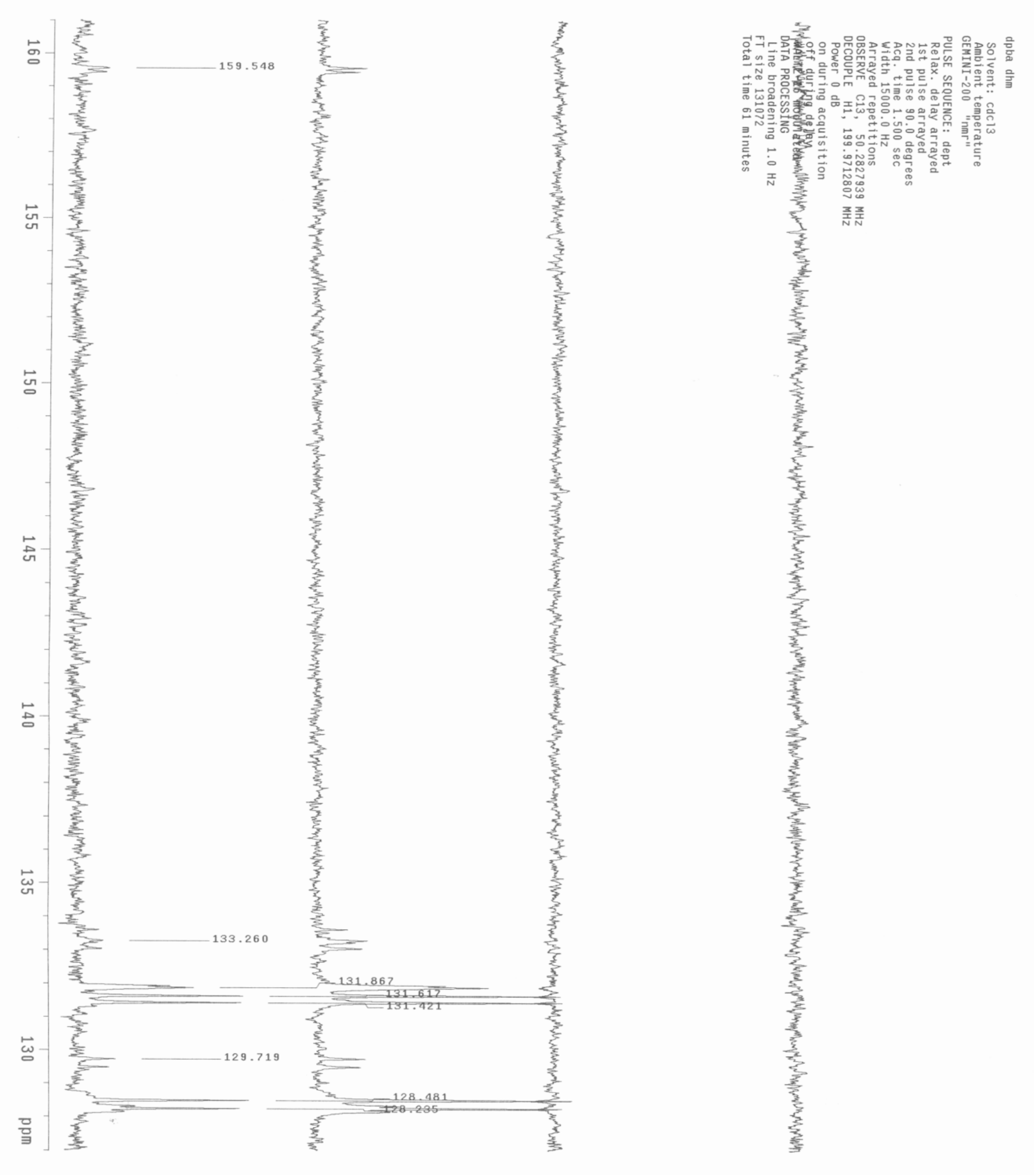

Figure S17. IR spectrum of compound **2**.

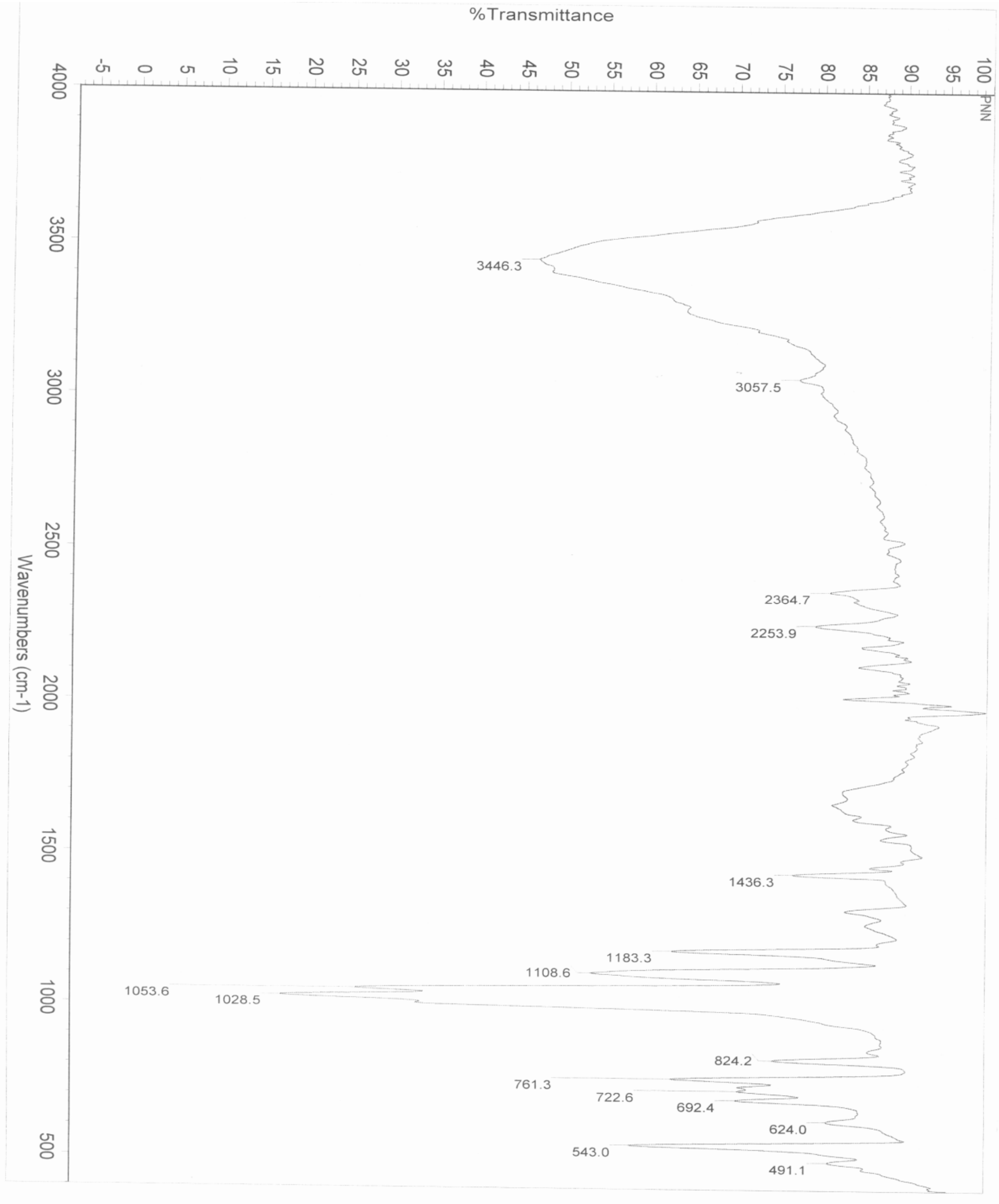

Supplement: Supplementary file 1 [file molecules-17-02567-s001.pdf]
